# A Novel Role of the PrpR as a Transcription Factor Involved in the Regulation of Methylcitrate Pathway in *Mycobacterium tuberculosis*

**DOI:** 10.1371/journal.pone.0043651

**Published:** 2012-08-16

**Authors:** Paweł Masiewicz, Anna Brzostek, Marcin Wolański, Jarosław Dziadek, Jolanta Zakrzewska-Czerwińska

**Affiliations:** 1 Department of Microbiology, Ludwik Hirszfeld Institute of Immunology and Experimental Therapy, Polish Academy of Sciences, Wrocław, Poland; 2 Laboratory of Mycobacterium Genetics and Physiology, Institute of Medical Biology, Polish Academy of Sciences, Łódź, Poland; 3 Department of Molecular Microbiology, Faculty of Biotechnology, University of Wrocław, Wrocław, Poland; Johns Hopkins University School of Medicine, United States of America

## Abstract

*Mycobacterium tuberculosis*, the pathogen that causes tuberculosis, presumably utilizes fatty acids as a major carbon source during infection within the host. Metabolism of even-chain-length fatty acids yields acetyl-CoA, whereas metabolism of odd-chain-length fatty acids additionally yields propionyl-CoA. Utilization of these compounds by tubercle bacilli requires functional glyoxylate and methylcitrate cycles, respectively. Enzymes involved in both pathways are essential for *M. tuberculosis* viability and persistence during growth on fatty acids. However, little is known about regulatory factors responsible for adjusting the expression of genes encoding these enzymes to particular growth conditions. Here, we characterized the novel role of PrpR as a transcription factor that is directly involved in regulating genes encoding the key enzymes of methylcitrate (methylcitrate dehydratase [PrpD] and methylcitrate synthase [PrpC]) and glyoxylate (isocitrate lyase [Icl1]) cycles. Using cell-free systems and intact cells, we demonstrated an interaction of PrpR protein with *prpDC* and *icl1* promoter regions and identified a consensus sequence recognized by PrpR. Moreover, we showed that an *M. tuberculosis prpR*-deletion strain exhibits impaired growth *in vitro* on propionate as the sole carbon source. Real-time quantitative reverse transcription-polymerase chain reaction confirmed that PrpR acts as a transcriptional activator of *prpDC* and *icl1* genes when propionate is the main carbon source. Similar results were also obtained for a non-pathogenic *Mycobacterium smegmatis* strain. Additionally, we found that *ramB*, a *prpR* paralog that controls the glyoxylate cycle, is negatively regulated by PrpR. Our data demonstrate that PrpR is essential for the utilization of odd-chain-length fatty acids by tubercle bacilli. Since PrpR also acts as a *ramB* repressor, our findings suggest that it plays a key role in regulating expression of enzymes involved in both glyoxylate and methylcitrate pathways.

## Introduction

Invasive pathogens, like *Mycobacterium tuberculosis*, subsist on nutrients from their host and persist for decades, ensuring effective transmission of bacteria. Accumulating evidence suggests a crucial role of fatty acids as a major carbon and energy source for this pathogen within host tissues. This usage pattern presumably reflects the increased availability of lipids in infected cells [1] and is consistent with the unique feature of the *M. tuberculosis* genome, which contains more than 200 genes involved in fatty acid degradation [2]. More recent reports describe the ability of *M. tuberculosis* to use cholesterol as a sole source of carbon and energy [3]–[5]. Cholesterol uptake and degradation processes appear to be essential for *M. tuberculosis* persistence in infected animals and growth within macrophages [3], [6]–[9]. Consistent with these observations, genes encoding β-oxidation enzymes, together with isocitrate lyase (*icl1*) and methylcitrate synthase (*prpC*), which are involved in anaplerotic metabolic cycles, are upregulated during infection of macrophages and mice, as are genes involved in cholesterol metabolism [10], [11].

Degradation of even-chain-length fatty acids leads to the formation of acetyl-CoA, whereas odd-chain-length fatty acid metabolism yields propionyl-CoA as an additional product. Degradation of the cholesterol side chain or ring structure also yields acetyl-CoA and propionyl-CoA [12]. These intermediates – acetyl-CoA and propionyl-CoA – are further metabolized via the glyoxylate and methylcitrate cycle, respectively [13]–[16]. When fatty acids or cholesterol are the sole carbon source for *M. tuberculosis*, these two metabolic pathways are necessary for bacterial survival. Interestingly, the product of the *icl1* (*rv0467*) gene in *M. tuberculosis* is involved in both glyoxylate and methylcitrate cycles, where it acts as an isocitrate lyase and methylisocitrate lyase, respectively [17]. However, the *prpDC* (*rv1130–1131*) operon encodes two additional enzymes – methylcitrate dehydratase (MCD) and methylcitrate synthase (MCS) – which are involved in the latter (methylcitrate) pathway. In contrast to *M. tuberculosis*, the *Mycobacterium smegmatis* genome contains the *prpB* gene, whose product exhibits methylcitrate lyase activity. In both *M. tuberculosis* and *M. smegmatis*, the *prpD(B)C* operon is essential for growth of mycobacteria on propionate as a sole carbon source *in vitro* [16], [18]. Additionally, the *M. tuberculosis* Δ*prpDC* strain is unable to propagate in murine macrophages. Metabolism of propionyl-CoA is important in another context: accumulated propionate as well as MCS/MCD-generated propionate metabolites are toxic and exert a dominant inhibitory effect on bacterial growth [18]. Thus, a functional methylcitrate cycle and isocitrate/methylisocitrate lyase activity are required for mycobacterial growth on propionate. Moreover, the fact that these enzymes (isocitrate lyase, methylcitrate dehydratase, and methylcitrate synthase) are absent in mammals makes them promising as potential drug targets. Little is currently known about the factors responsible for regulating *icl1* or *prpDC* expression during *M. tuberculosis* growth under different conditions. RamB (Rv0465c) was recently characterized as a transcription factor that binds the *icl1* promoter region and represses expression of this gene during *M. tuberculosis* growth on glucose as a major carbon source [19]. RamB also binds its own promoter and negatively autoregulates its expression.

**Figure 1:**
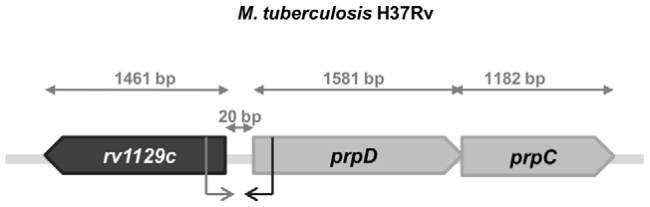
Schematic depiction of the *rv1129* (*prpR*) region on the *M. tuberculosis* H37Rv chromosome. *prpD* and *prpC* genes encode methylcitrate dehydratase and methylcitrate synthase, respectively. The bent arrow represents the putative transcription start sites of *prpR* (see Fig. S6) and *prpD* genes [21].

In order to identify a previously undiscovered regulatory factor that might be involved in the regulation of glyoxylate and/or methylcitrate cycles, we thoroughly analyzed the *M. tuberculosis* H37Rv genome sequence. Interestingly, we found that open reading frame Rv1129c, which encodes a putative transcriptional regulator, is located in close proximity to the *prpDC* operon (20 bp from the *prpD* start codon; see Figure 1). Additionally, the orientations of *rv1129c* and *prpDC* transcription are opposite, strongly suggesting that they possess a common promoter region. Therefore, we hypothesized that Rv1129c may act as a regulator of the *prpDC* gene as well as an autoregulator of its own expression. Interestingly, global gene expression analyses revealed that the *rv1129c* gene is important for *M. tuberculosis* pathogenesis. Moreover, *rv1129c* was among the genes found to be essential for *M. tuberculosis* survival in the mouse hollow-fiber model of hypoxia [20]. It was subsequently shown that expression of *rv1129c* and *prpDC* genes, as well as that of *icl1*, is activated by the sigma-E (σ^E^) subunit of RNA polymerase in response to detergent-mediated, cell-surface stress [21]. Additionally, we determined that the *msmeg_6643* gene – the *rv1129c* ortholog in *M. smegmatis* mc^2^ 155– is also located directly adjacent to the *prpDBC* operon. In this case, the intergenic region contains 108 bp, and the probable regulatory gene and operon are also transcribed in opposite directions. Thus, we concluded that the putative role of *rv1129c*/*msmeg_6643* gene products in the regulation of methylcitrate enzymes expression may be conserved in both mycobacterial species. Rv1129c and Msmeg_6643 proteins share 78% similarity and 68% sequence identity and have molecular masses of about 55 and 52 kDa, respectively. They belong to the XRE (Xenobiotic Response Element) family of transcriptional regulators and contain a helix-turn-helix (HTH_3) DNA-binding motif at their N-terminus. Members of this family are not well characterized, but in bacteria, some XRE regulators are involved in stress responses [22].

In the present work, we describe the interaction of Rv1129c protein with *icl1*, *prpDC* and *ramB* promoter regions, and characterize its role in the regulation of these genes' expression during *M. tuberculosis* growth in media containing different carbon sources. We also show that the level of *rv1129c* expression is highest in media containing products of odd-chain-length catabolism (e.g., propionate) as a carbon source. Therefore, we name the *rv1129c*/*msmeg_6643* locus, *prpR* (propionate regulator). Interestingly, we also found that PrpR protein interacts with the promoter region of *kstR* (*rv3574*) gene which is involved in the transcriptional regulation of cholesterol utilization in *M. tuberculosis* [23], [24]. Therefore, we hypothesized that PrpR may play a role also during growth of the tubercle bacilli in the cholesterol-containing environment.

## Results

### *M. tuberculosis* PrpR protein binds the promoter region of *prpDC* and *icl1* genes

**Figure 2:**
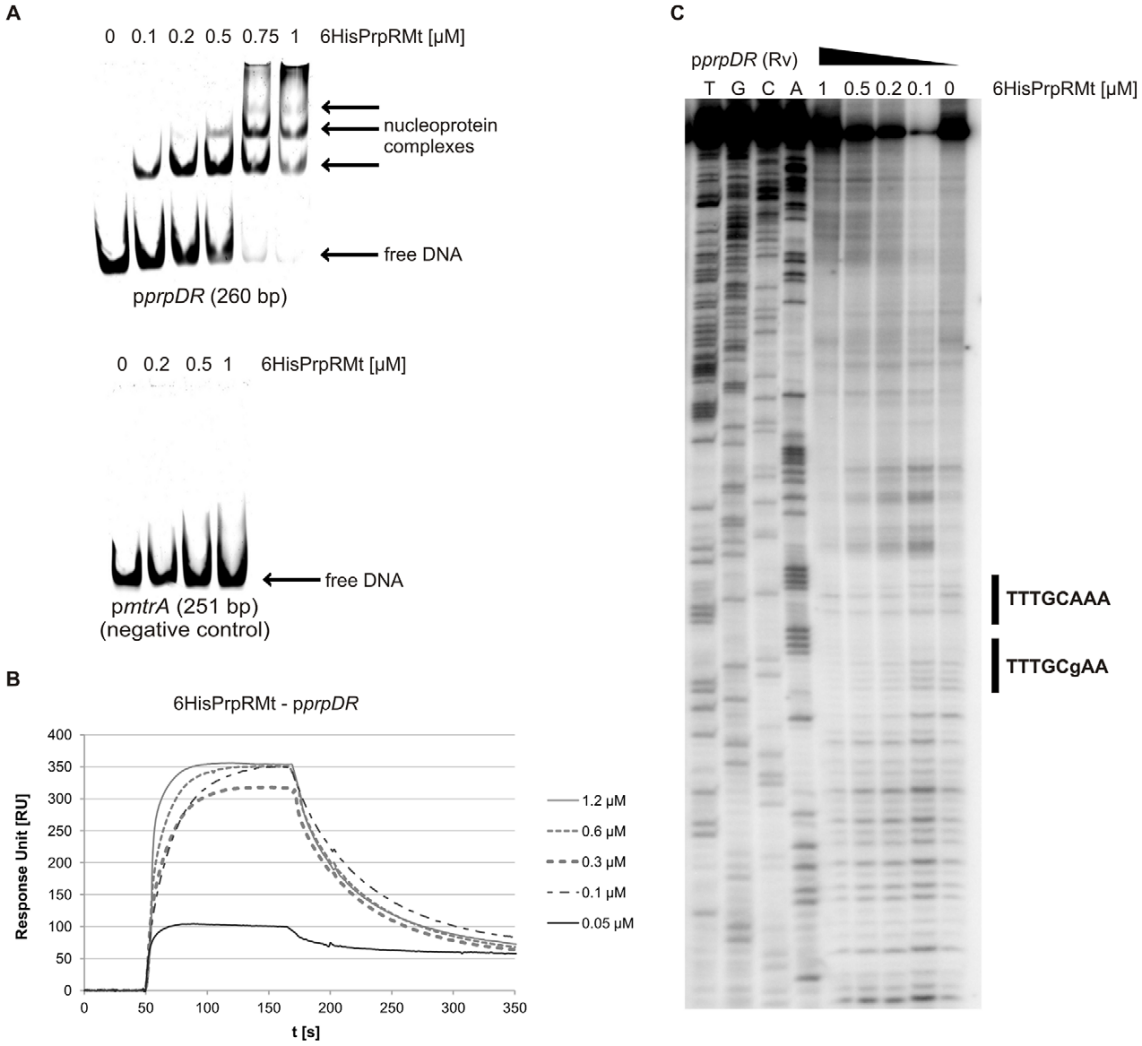
PrpR interacts with the promoter region of the *prpD* gene. (A) EMSA. Oligonucleotides corresponding to the *prpD* (260 bp) or *mtrA* (251 bp, negative control) promoter region were incubated with increasing amounts of 6HisPrpRMt protein; nucleoprotein complexes were analyzed on 4% polyacrylamide gels. (B) SPR. Sensograms were obtained by binding 6HisPrpRMt to biotinylated DNA fragment corresponding to the promoter region of the *prpD* gene (p*prpDR*, 260 bp), amplified with biotinylated p1129_Fw and p1129_Rv primers and immobilized on a streptavidin-coated chip in the BIAcore apparatus. (C) DNase I footprinting. A ^32^P-labeled, 260-bp DNA fragment was incubated with increasing amounts of 6HisPrpRMt protein and then subjected to DNase I digestion. Lanes: T, G, C, and A represent sequencing reactions for the p*prpRD* fragment. Radiolabeled p1129_Rv primer was used to perform sequencing reactions.

In order to establish the role of the *rv1129c*-encoded *M. tuberculosis* PrpR protein (PrpRMt) in the regulation of *prpDC* transcription, we first sought to determine whether this protein interacts with the *prpDC* promoter region. For this purpose, we purified PrpRMt as an N-terminal 6His-tagged fusion protein (6HisPrpRMt), and then characterized the interaction of 6HisPrpRMt with a 260-bp DNA fragment containing a common *prpD* and *prpR* promoter region (p*prpDR*, see Figure S6) in cell-free systems using electrophoretic mobility shift assays (EMSAs) and surface plasmon resonance (SPR). In EMSAs, increasing amounts of the recombinant protein clearly retarded p*prpDR* fragment migration in polyacrylamide gels (Figure 2A). A specific band was observed in the presence of 6HisPrpRMt at concentrations as low as 0.1 µM, whereas additional bands appeared at protein concentrations of 0.5–0.75 µM; at the highest 6HisPrpRMt concentration (1 µM), no bands migrating as free DNA were visible. In contrast, we observed no binding of 6HisPrpRMt to the DNA fragment p*mtrA*, used as a negative control. An interaction of 6HisPrpRMt with p*prpDR* was also confirmed by SPR (Figure 2B), which showed that response unit (RU) values were proportional to protein concentration (0.05–1.2 µM). Interestingly, cross-linking and bacterial two-hybrid assays revealed that PrpRMt forms stable dimers and possibly trimers (Figure S1). Therefore, we hypothesize that PrpRMt, like many other transcriptional regulators, may interact with DNA as a dimer.

**Figure 3:**
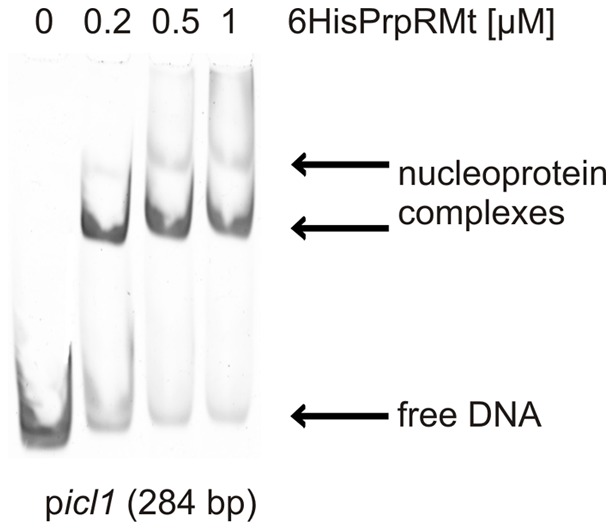
PrpR interacts with the promoter region of the *icl1* gene (EMSA). The *icl1* (284 bp) promoter region was incubated with increasing amounts of 6HisPrpRMt protein; nucleoprotein complexes were analyzed on 4% polyacrylamide gels.

The PrpRMt binding site within the *prpDR* promoter region was identified by DNase I footprinting. In the presence of increasing amounts of 6HisPrpRMt, a relatively long stretch of DNA (25 bp) was protected against nuclease digestion. Interestingly, two 8-bp palindromic sequences separated by a single nucleotide – one perfect (TTTGCAAA) and one imperfect (TTTGCgAA) – were identified within the protected region (Figure 2C), suggesting that this motif might be the PrpR binding site. To determine whether the identified PrpRMt binding motif is present within promoter regions of other genes, we performed a global search of the *M. tuberculosis* H37Rv genome using the Fuzznuc application (http://anabench.bcm.umontreal.ca/anabench/Anabench-Jsp/Applications/fuzznuc.jsp). This database analysis identified 15 perfect TTTGCAAA palindromic sequences within the entire *M. tuberculosis* H37Rv genome, six of which were located in putative promoter regions (Tables S1 and S2). Most interestingly, the one perfect TTTGCAAA palindrome identified by DNase I footprinting was located in the *icl1* promoter, 175 bp upstream of the ATG codon. Using an EMSA assay to test the interaction of the 6HisPrpRMt protein with a DNA fragment (284 bp) containing the *icl1* promoter region (p*icl1*), we found that 6HisPrpRMt strongly retarded p*icl1* fragment migration in a polyacrylamide gel (Figure 3). A specific band was detected in the presence of 0.2 µM protein, and additional bands appeared with increasing 6HisPrpRMt concentration (up to 1 µM). Thus, EMSA confirmed strong 6HisPrpRMt-p*icl1* interaction.

**Figure 4:**
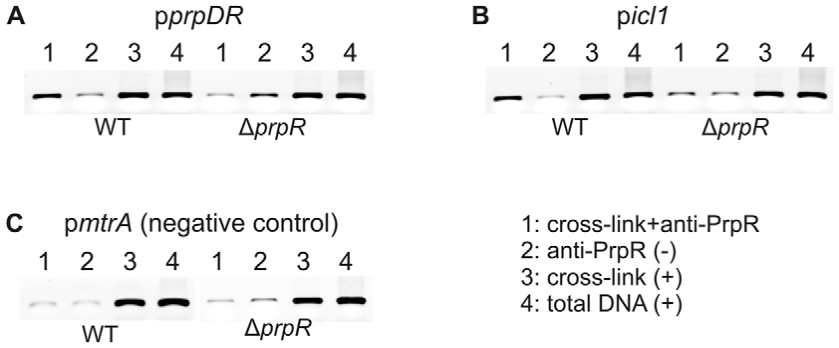
PrpR binds the promoter region of *prpDC* and *icl1* genes in intact *M. tuberculosis* cells. Identification of intracellular PrpR-DNA complex using immunoprecipitation. PrpR-DNA complexes cross-linked with glutaraldehyde were immunoprecipitated with anti-6HisPrpRMt polyclonal antibodies (sample 1). PCR was carried out with the primer pairs, p1129_Fw and p1129_Rv (p*prpDR*)(A); picl_Fw and picl_Rv (p*icl1*)(B); and pmtrA_Fw and pmtrA_Rv (p*mtrA*, negative control)(C). Negative control (2) consisted of DNA template extracted from the cells subjected to immunoprecipitation, but nucleoprotein complexes were not previously cross-linked. Positives controls (+) were also performed using template obtained from strains subjected only to cross-linking (3) or total DNA extracted from the cells (4).

To determine whether the PrpRMt regulator is also able to bind *prpDR* and *icl1* gene promoter regions in *M. tuberculosis* cells, we performed immunoprecipitation assays using affinity chromatography-purified antibodies against the PrpRMt protein, raised in rabbits. The cross-linked PrpRMt-DNA complexes formed in the *M. tuberculosis* H37Rv wild-type strain were enriched by affinity chromatography, and released DNA fragments were identified by conventional polymerase chain reaction (PCR) using primers specific for *prpDR* and *icl1* promoter regions; a DNA fragment that is not a PrpRMt target was used as a negative control. An *M. tuberculosis prpR*-deletion strain (Δ*prpR*) served as an additional negative control. We obtained strong PCR signals in reactions with both p*prpDR* ***-*** and p*icl1*-specific primers, but only in DNA samples derived from *M. tuberculosis* wild-type strain subjected to cross-linking followed by anti-PrpRMt antibody precipitation (Figure 4A and 4B, the negative controls displayed a faint background signal). Thus, immunoprecipitation confirmed the interaction of PrpRMt regulator with the analyzed promoter regions within intact *M. tuberculosis* cells.

### PrpRMt interacts with the promoter region of the *ramB* gene, involved in the regulation of the glyoxylate cycle

**Figure 5:**
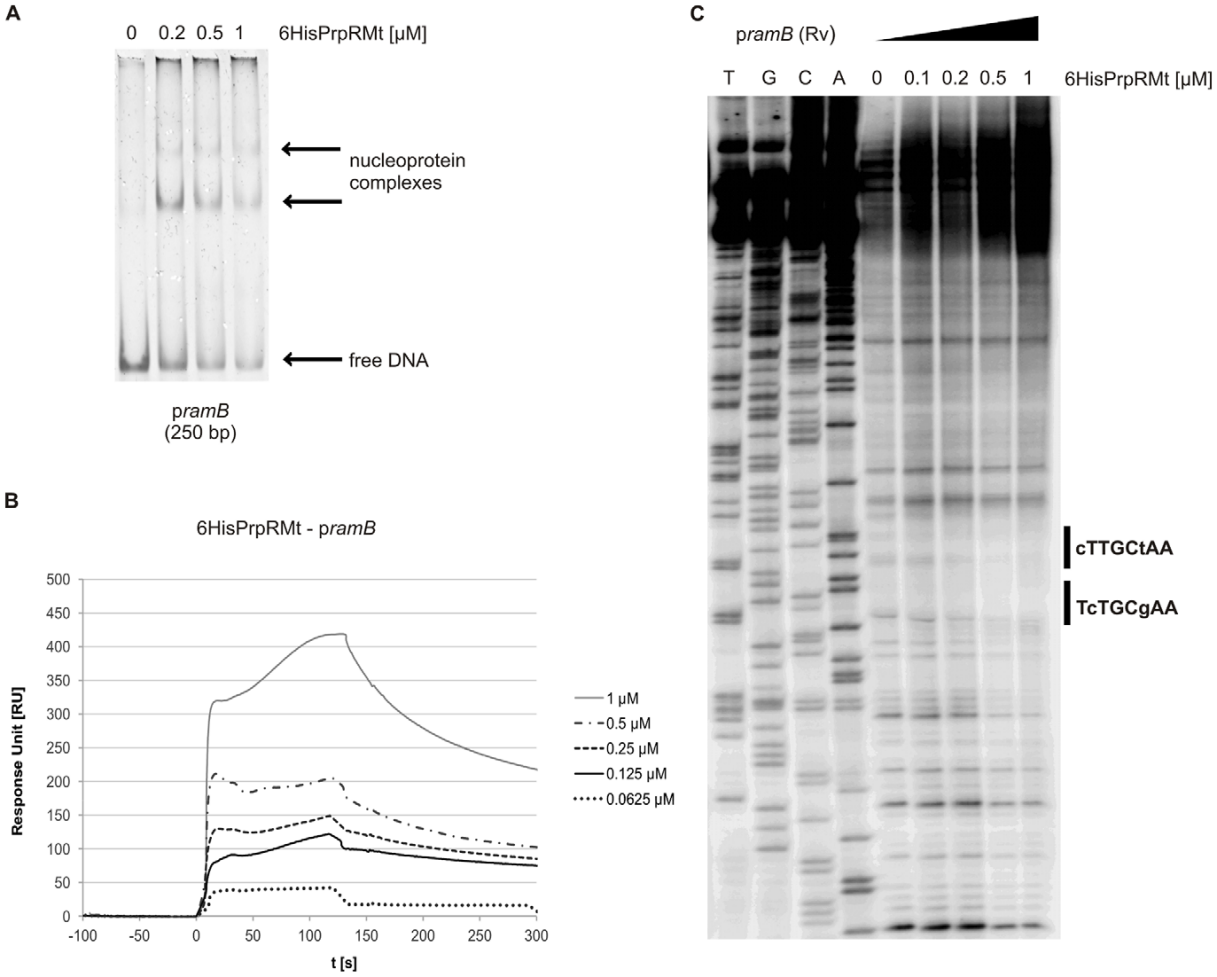
PrpR interacts with the promoter region of the *ramB* gene. (A) EMSA. An oligonucleotide (250 bp) corresponding to the *ramB* promoter region was incubated with increasing amounts of 6HisPrpRMt protein; nucleoprotein complexes were analyzed on 4% polyacrylamide gels. (B) SPR. Sensograms were obtained by binding 6HisPrpRMt to a biotinylated DNA fragment (250 bp) corresponding to the promoter region of the *ramB* gene (p*ramB*), amplified with biotinylated pramB_Fw and pramB_Rv primers and immobilized on a streptavidin-coated chip in the BIAcore apparatus. (C) DNase I footprinting. A ^32^P-labeled, 250 bp DNA fragment was incubated with increasing amounts of 6HisPrpRMt protein and then subjected to DNase I digestion. Lanes: T, G, C, and A represent sequencing reactions for the p*ramB* fragment. Radiolabeled pramB_Rv primer was used to perform sequencing reactions.

Interestingly, it has been recently shown that, like PrpR, the PrpR paralog RamB, which also belongs to the XRE family of transcription factors, interacts with the promoter regions of the *icl1* gene as well as that of its own gene. Micklinghoff *et al*. [19] demonstrated that RamB is involved in the regulation of the glyoxylate cycle; it represses *icl1* expression during growth with glucose and negatively autoregulates the expression of its own gene. Because RamB and PrpR are paralogs, sharing 65% similarity and 48% sequence identity [25], we hypothesized that the *ramB* promoter region (p*ramB*) may also be a PrpRMt target, and *vice versa*. It should be noted that neither the previously identified perfect palindrome (TTTGCAAA) nor the single mismatched palindrome are present in the *ramB* promoter region. However, we did find two imperfect palindromes separated by a single nucleotide, cTTGCtAA and TcTGCgA, in this region, each with two mismatches. Using EMSA and SPR to investigate the interaction of PrpR and RamB proteins with the corresponding heterologous promoter, we found that both techniques revealed strong binding (K_D_ = 32 nM) of 6HisPrpRMt to a DNA fragment (250 bp) containing the *ramB* promoter (Figure 5A and 5B). However, we did not observe an interaction between 6HisRamB and p*prpDR* (Figure S2). Moreover, in contrast to PrpR protein, RamB bound its own promoter with a rather weak affinity (K_D_ = 328 nM), consistent with a previous report by Micklinghoff *et al*. [19].

DNase I footprinting analyses showed that 6HisPrpRMt weakly protected the p*ramB* region containing the two imperfect palindromes from nuclease digestion (Figure 5C). As expected, 6HisRamB afforded no protection from nuclease digestion within this region (data not shown). Taken together, our results demonstrated that PrpRMt exhibits a higher affinity than RamB for the *ramB* promoter region.

### Deletion of the *prpR* gene results in impaired growth of *M. tuberculosis* on propionate as a sole carbon source

**Figure 6:**
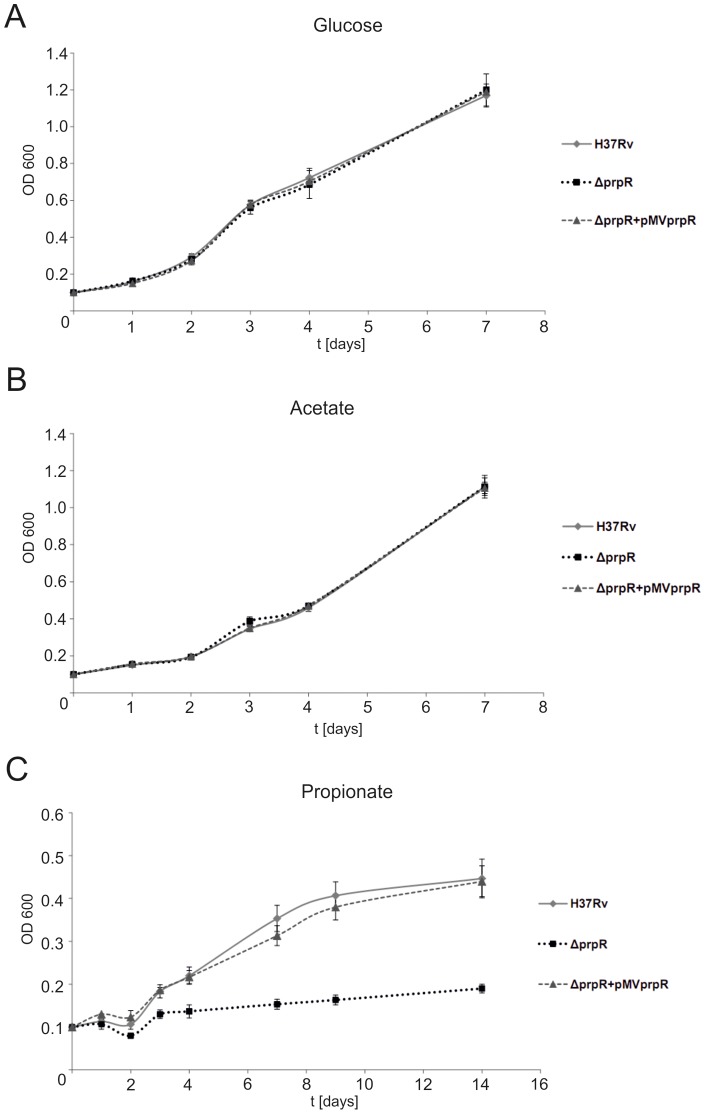
The *prpR*-deletion mutant of *M. tuberculosis* H37Rv exhibits impaired growth on propionate as a sole carbon source. Comparison of wild-type (H37Rv), Δ*prpR* (*prpR*-deletion mutant) and Δ*prpR+*pMV*prpR* (complementation of Δ*prpR* strain) strains of *M. tuberculosis* H37Rv, grown on different carbon sources. Growth was monitored in M9 minimal medium supplemented with glucose (A), acetate (B), or propionate (C) (0.5% each). Wild-type and deletion strain growth curves were significantly different (p<0.05), but wild-type and complementation strain growth curves were similar (p>0.05).

The interaction of PrpRMt with the promoter region of *icl1*, *prpDC*, and *ramB* genes strongly suggested the potential involvement of PrpRMt protein in the regulation of genes responsible for utilization of fatty acid β-oxidation products. In order to determine the effect of *prpR* deletion on utilization of these compounds by tubercle bacilli, we constructed the *M. tuberculosis* Δ*prpR* strain. The *prpRmt* (*rv1129c*) gene was disrupted in such a way that the resulting mutant strain lacked the DNA fragment encoding the HTH_3 motif, but retained the σ^E^-dependent *prpDC* promoter (168 bp downstream of the *prpRmt* start codon). The deletion was confirmed by PCR and Southern blotting (Figures S3 and S4). Next, we analyzed the growth rate of *M. tuberculosis* strains in media containing different carbon sources (glucose, acetate, or propionate). As expected, we did not observe a significant difference in growth between *M. tuberculosis* H37Rv wild-type and Δ*prpR* strains in medium containing glucose (Figure 6A). Surprisingly, *prpR* deletion also had no effect on the utilization of acetate as a sole carbon source by tubercle bacilli (Figure 6B). However, the growth of the *M. tuberculosis* Δ*prpR* strain was significantly impaired in medium containing propionate as the major carbon source. After 2 weeks of cultivation, the deletion strain achieved an optical density (OD) of 0.2, whereas that for the wild-type strain was almost 0.5 (Figure 6C). Normal growth on propionate was restored to the Δ*prpR* strain by complementation with a single-copy integrating plasmid containing the *prpR* gene under the control of its own promoter. Therefore, we conclude that the *prpR* gene is indispensable for *M. tuberculosis* growth *in vitro* on propionate as a sole carbon source.

### The *prpR* gene is most highly expressed during *M. tuberculosis* growth on propionate

**Figure 7:**
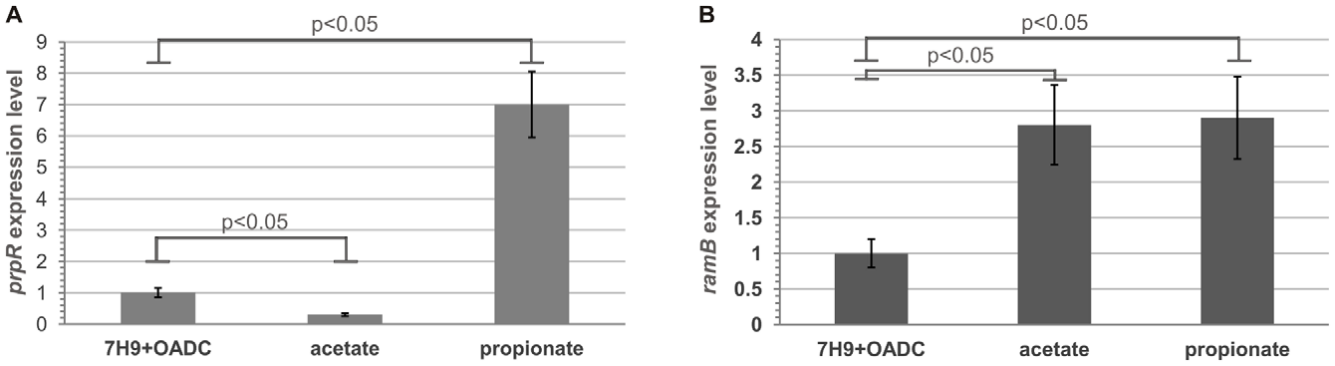
Influence of carbon source on the expression of *prpR* and *ramB* genes in *M. tuberculosis* H37Rv. Total RNA was extracted from a culture growing in 7H9+OADC broth (rich medium) or M9 minimal medium containing either acetate or propionate (0.5%) until reaching an OD_600_ value of 0.6–0.8. For minimal media, cells were incubated for an additional 48 hours. The primer pairs for analysis of genes by qPCR are listed in Table S5. mRNA levels of target genes, *prpR* (A), *ramB* (B), were normalized internally to that of the constitutively expressed housekeeping gene, *sigA*. Means were calculated from three independent experiments and three determinations per experiment. The error bars indicate standard deviations of triplicate samples. Statistical significance was calculated by the Student's t-test.

Since the presence of the *prpR* gene appeared to be crucial for the growth of *M. tuberculosis* on propionate (a degradation product of odd-chain-length fatty acids), but not on media containing glucose or acetate, we postulated that the expression of this gene might be affected by the carbon source. To investigate this hypothesis in detail, we used quantitative real-time RT-PCR analysis to analyze cDNA templates derived from the *M. tuberculosis* H37Rv strain cultivated in rich medium containing glucose (7H9+OADC) and in M9 minimal medium containing either acetate or propionate as a sole carbon source. As predicted, *prpR* expression was highest in *M. tuberculosis* grown on propionate; under these conditions, *prpR* expression was approximately 7-times higher than that in *M. tuberculosis* grown on rich medium (7H9+OADC broth) (Figure 7A). Interestingly, the lowest *prpR* expression level was observed during *M. tuberculosis* growth in medium containing acetate as a carbon source. This value was approximately 4-times lower than that in 7H9+OADC medium and almost 30-times lower compared to that in minimal medium with propionate. Therefore, qPCR confirmed that *prpR* expression is directly dependent on the carbon source and significantly increases during *M. tuberculosis* growth on products derived from β-oxidation of odd-chain-length fatty acids. However, since *ramB* is a *prpR* paralog, it was necessary to examine whether its expression is also carbon source-dependent. Additional qPCR analyses showed that *ramB* expression levels in media containing acetate or propionate as a sole carbon source were comparable and were approximately 3-times higher than those in *M. tuberculosis grown* in 7H9+OADC broth (Figure 7B). These results strongly suggest that utilization by tubercle bacilli of products obtained during even- or odd-chain-length fatty acid catabolism may be subjected to control by different transcription factors belonging to the XRE family.

### PrpRMt activates transcription of *prpDC* and *icl1* genes and represses *ramB* during growth on propionate

**Figure 8:**
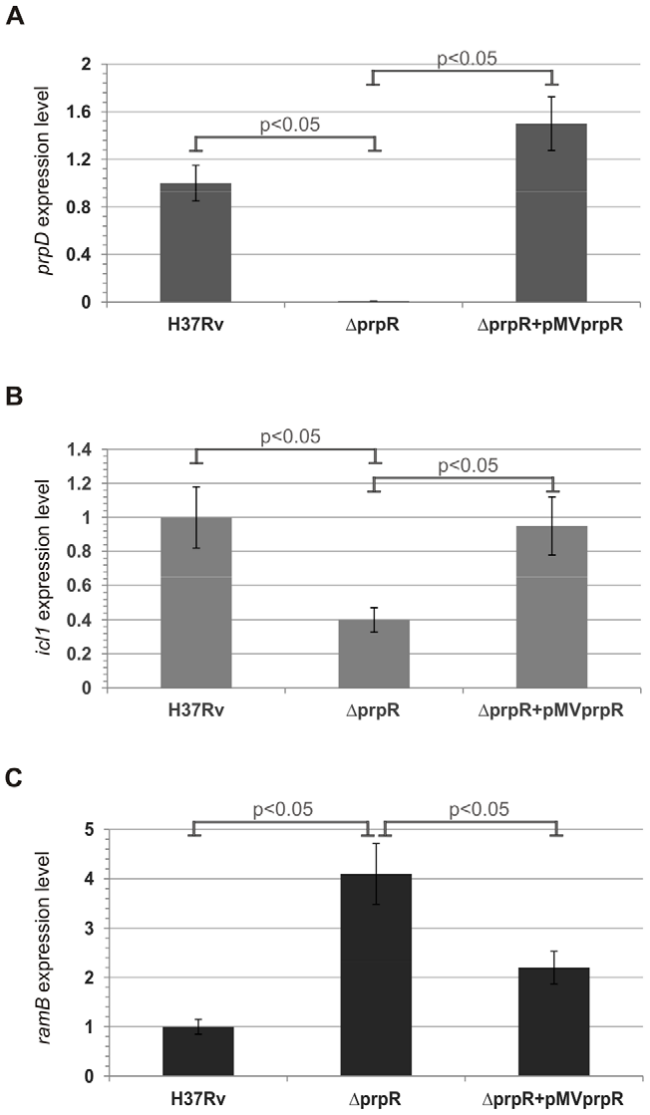
PrpR promotes transcription of *prpDC* and *icl1* and represses *ramB* during growth on propionate. Indicated *M. tuberculosis* strains were grown in M9 minimal medium containing propionate and total RNA was extracted. The primer pairs for analysis of genes by qPCR are listed in Table S5. mRNA levels of target genes, *prpDC* (A), *icl1* (B), and *ramB* (C) were normalized internally to that of the constitutively expressed housekeeping gene, *sigA*. Means were calculated from three independent experiments and three determinations per experiment. The error bars indicate standard deviations of triplicate samples. Statistical significance was calculated by the Student's t-test.

**Figure 9:**
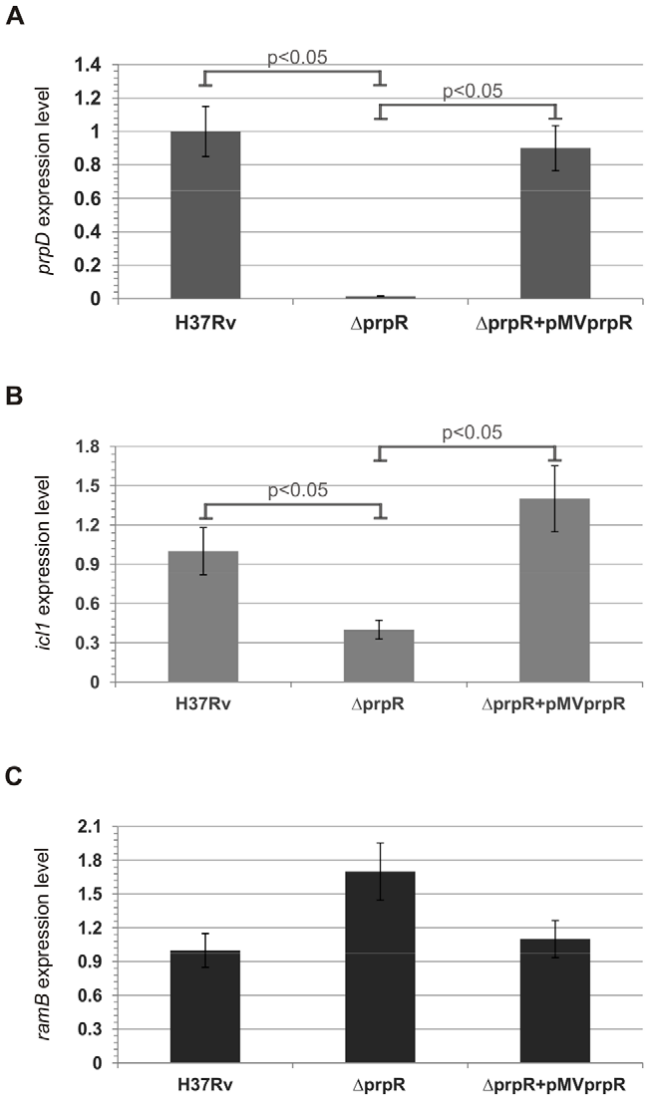
PrpR promotes transcription of both *prpDC* and *icl1* in *M. tuberculosis* during growth in rich 7H9+OADC broth, but does not influence *ramB* expression. Analyzed *M. tuberculosis* strains were grown in 7H9+OADC broth (rich medium) until reaching an OD_600_ value of 0.6–0.8 and total RNA was extracted. The primer pairs for analysis of genes by qPCR are listed in Table S5. mRNA levels of target genes, *prpDC* (A), *icl1* (B), and *ramB* (C) were normalized internally to *sigA*. Means were calculated from three independent experiments and three determinations per experiment. The error bars indicate standard deviations of triplicate samples. Statistical significance was calculated by the Student's t-test.

In order to more precisely understand the role of the PrpRMt transcription factor in the regulation of genes involved in the glyoxylate and methylcitrate cycles, we performed qRT-PCR analysis using RNA isolated from *M. tuberculosis* strains cultivated in media containing different carbon sources. We measured the expression levels of *prpDC*, *icl1* and *ramB* genes in *M. tuberculosis* H37Rv wild-type, Δ*prpR,* and complemented (Δ*prpR*+pMV*prpR*) strains grown on 7H9+OADC (rich medium) and M9 minimal medium containing either acetate or propionate as a sole carbon source. As expected, PrpRMt exerted its strongest regulatory effects on *prpDC*, *icl1*, and *ramB* expression when strains were cultivated on propionate. Indeed, *prpD* and *icl1* expression levels in the deletion mutant were 100- and 3-fold lower, respectively, than in the wild-type (Figure 8A and 8B). The effects of PrpRMt on the *prpC* gene (almost a 100-fold decrease in expression level; data not shown) were similar to those on *prpD*, suggesting that both genes belong to the same transcriptional unit – operon (the genes are separated by only two nucleotides). In contrast, the level of *ramB* expression was found to be almost 4-fold higher in the Δ*prpR* strain than in the wild-type *M. tuberculosis* strain (Figure 8C). In all three cases (*prpDC*, *icl1*, and *ramB*), a wild-type-like expression level was restored in the complemented strain, confirming that the identified regulatory effect was directly dependent on PrpRMt. These results clearly demonstrate the essential role of PrpRMt in activation of the *prpDC* operon and induction of the *icl1* gene, as well as repression of *ramB,* during *M. tuberculosis* growth in medium containing products of odd-chain-length fatty acid catabolism. Similar effects on *prpDC* and *icl1* expression were also obtained when analyzed strains were grown in rich 7H9+OADC broth. Under these conditions, the expression levels of *prpD* and *icl1* were almost 100- and 3-fold lower, respectively, in the deletion mutant than in the wild-type (Figure 9A and 9B). Although expression of the *ramB* gene trended higher (∼1.7-fold) in the *M. tuberculosis* Δ*prpR* strain than in the wild-type H37Rv strain in rich media, this difference did not reach statistical significance (Figure 9C); thus, whether PrpRMt also regulates *ramB* expression in these conditions is not entirely clear. Nevertheless, the appropriate expressions of all analyzed genes were restored in the complemented *M. tuberculosis* Δ*prpR*+pMV*prpR* strain. Therefore, these results also demonstrate the role of PrpRMt as a direct transcriptional activator of *prpD* and *icl1* in *M. tuberculosis* grown in rich medium *in vitro*. We also analyzed the expression levels of *prpD*, *icl1*, and *ramB* genes in *M. tuberculosis* strains cultivated on acetate as a sole carbon source. Notably, we did not identify any significant differences in the expression of these genes in the deletion mutant compared to the wild-type (data not shown). Therefore, we conclude that PrpRMt is not likely involved in the regulation of gene expression during *M tuberculosis* growth on medium containing products of even-chain-length fatty acid β-oxidation.

### PrpR binds promoter region of *kstR* and activates its transcription during growth on rich medium

**Figure 10:**
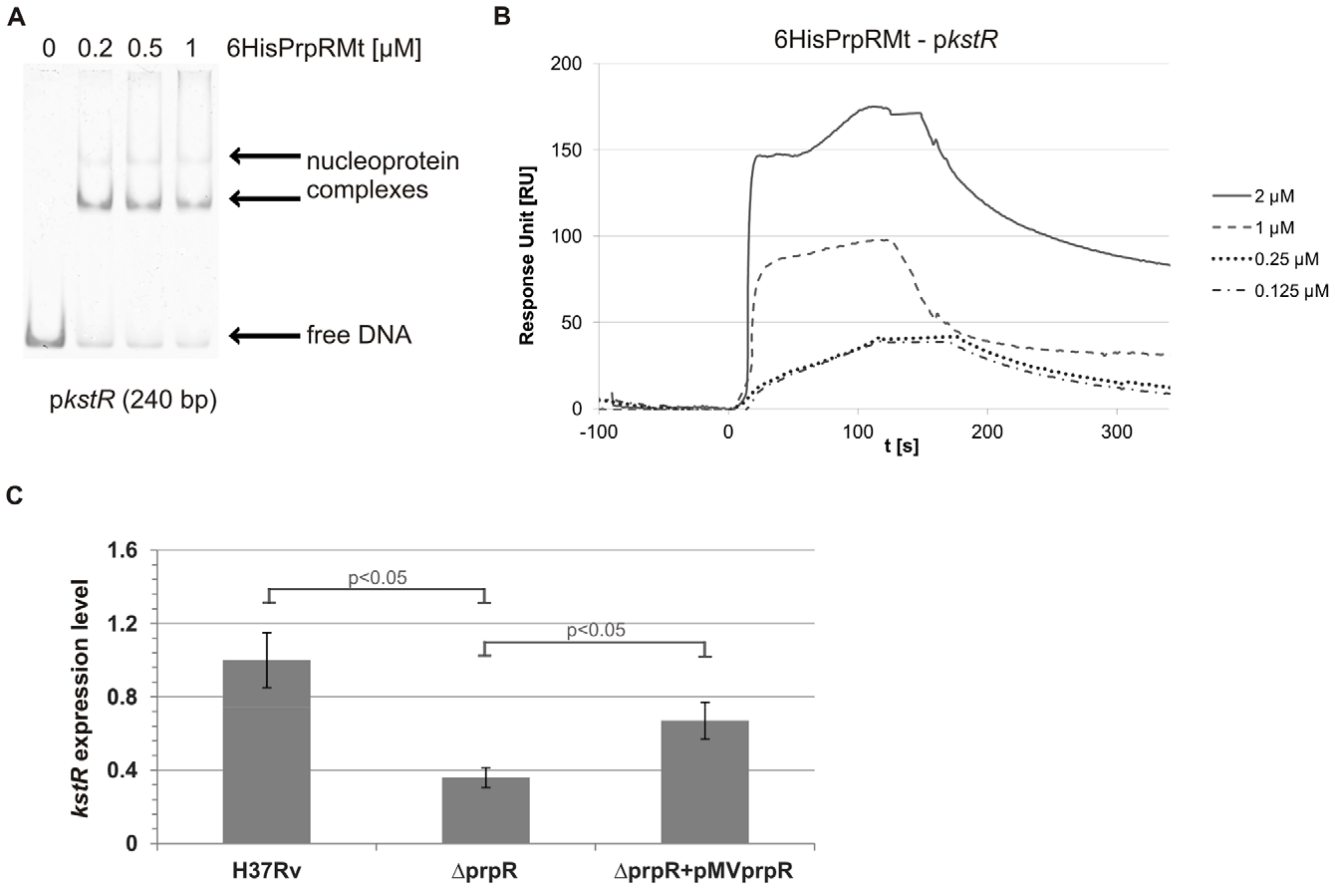
PrpR interacts with the promoter region of the *kstR* gene and activates its transcription during *M. tuberculosis* growth in rich medium. (A) EMSA. An oligonucleotide (240 bp) corresponding to the *kstR* promoter region was incubated with increasing amounts of 6HisPrpRMt protein; nucleoprotein complexes were analyzed on 4% polyacrylamide gels. (B) SPR. Sensograms were obtained by binding 6HisPrpRMt to a biotinylated DNA fragment (240 bp) corresponding to the promoter region of the *kstR* gene (p*kstR*), amplified with biotinylated pkstR_Fw and pkstR_Rv primers and immobilized on a streptavidin-coated chip in the BIAcore apparatus.(**C**) qPCR. Total RNA was extracted from indicated *M. tuberculosis* strains growing in 7H9+OADC broth (rich medium). mRNA levels of *kstR* gene were normalized internally to *sigA*. Means were calculated from three independent experiments and three determinations per experiment. The error bars indicate standard deviations of triplicate samples. Statistical significance was calculated by the Student's t-test.

Interestingly, the one prefect TTTGCAAA palindrome was also identified in the *kstR* (*rv3574*) promoter, 102 bp upstream of the GTG start codon (Table S1). Using EMSA and SPR to investigate the interaction of the 6HisPrpRMt protein with a DNA fragment (240 bp) containing the *kstR* promoter, we found that both techniques revealed strong binding (K_D_ = 25 nM) of 6HisPrpRMt to this promoter (Figure 10A and 10B). This finding suggested the potential involvement of PrpRMt protein in the regulation of *kstR* expression and, consequently, controlling the cholesterol catabolism in *M. tuberculosis*. However, we did not observe a significant difference in the growth between *M. tuberculosis* H37Rv wild-type and Δ*prpR* strains in medium containing cholesterol as the major carbon source; after 7 days of cultivation, the deletion strain achieved an OD_600_ of 0.4, whereas that for the wild-type strain was 0.6 (data not shown). Additionally, by using qPCR analysis with cDNA templates derived from *M. tuberculosis* strains growing on propionate as a sole carbon source, we did not identify influence of *prpR* deletion on *kstR* expression (data not shown). Interestingly, *kstR* expression level on the rich 7H9+OADC broth was decreased (almost 3-fold) in the Δ*prpR* mutant compared to the wild-type strain. The wild-type expression level of *kstR* was partially restored in the complemented *M. tuberculosis* Δ*prpR*+pMV*prpR* strain (Figure 10C). These results confirmed our initial hypothesis that PrpR is possibly involved in the regulation of genes responsible for controlling cholesterol utilization by the tubercle bacilli.

## Discussion

**Figure 11:**
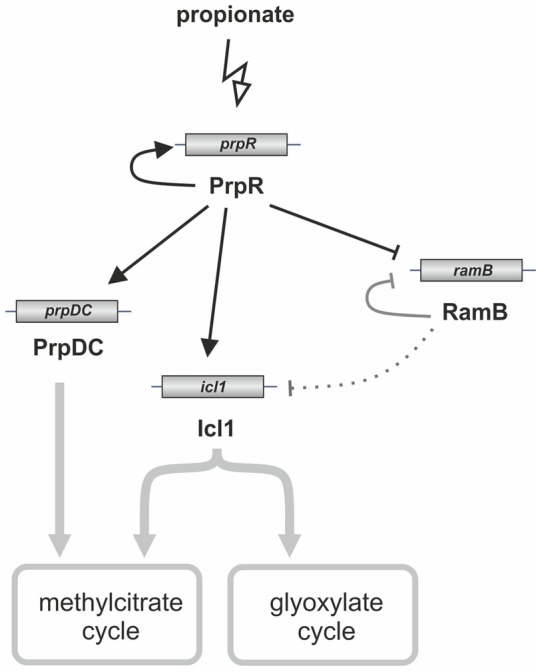
PrpR regulates the expression of genes involved in methylcitrate and glyoxylate cycles in *M. tuberculosis*. Perpendicular lines indicate negative regulation. Grey perpendicular lines from Micklinghoff *et al*. [11].

Metabolism of fatty acids plays a crucial role in *M. tuberculosis* persistence within the human host. When lipids provide the major carbon source for bacteria, glyoxylate and methylcitrate cycles are responsible for utilization of acetyl-CoA and propionyl-CoA, derived from catabolism of even- and odd-chain-length fatty acids, respectively. ICL1, encoded by the i*cl1* gene, exhibits isocitrate lyase as well as methylisocitrate lyase activity and is the key enzyme required for both cycles [17]. Previous studies have indicated that methylcitrate dehydratase and methylcitrate synthase, involved in the methylcitrate cycle, are essential for the growth of tubercle bacilli on fatty acids *in vitro* and in macrophages [15], [16]. Moreover, expression of *icl1* and *prpC*, encoding methylcitrate dehydratase and methylcitrate synthase, respectively, is also significantly increased during mouse lung infection [11], [26] as well as in macrophages [10], supporting the hypothesis that *M. tuberculosis* subsists on fatty acids within the host. However, little is known about the regulatory mechanisms that control the expression of these genes. RamB (Rv0465c) was recently described as a transcription factor involved in the control of the glyoxylate cycle in *M. tuberculosis* [19]. In addition to binding its own promoter, RamB interacts with the *icl1* promoter region and represses *icl1* expression during *M. tuberculosis* growth on glucose. In the present study, we demonstrated the novel role of PrpR (Rv1129c) as a transcription factor involved in the regulation of both methylcitrate and glyoxylate cycles (Figure 11).

The *prpR* (*rv1129c*) gene is located adjacent to the *prpDC* (*rv1130–1131*) operon in the *M. tuberculosis* H37Rv chromosome. The *prpR* and *prpD* genes are separated by only 20 bp and are transcribed in opposite directions (Figure 1), strongly suggesting the potential involvement of PrpR in regulating the expression of its own gene and that of *prpDC*. Indeed, PrpRMt specifically recognized an 8-bp palindromic sequence, TTTGCAAA, within the *prpD-prpR* region (Figure 2) and interacted with this region in *M. tuberculosis* cells (Figure 4). Interestingly, we also found a putative PrpR binding site (perfect palindrome) within the promoter region of the *icl1* (*rv0467*) gene and demonstrated that PrpR interacted with this region in cell-free systems and intact *M. tuberculosis* cells (Figures 3 and 4).

Because PrpR and RamB are paralogs (65% similarity) and both proteins bound not only their own promoter but also the *icl1* promoter region, we reasoned that they might reciprocally regulate each other's expression as well as that of *icl1*. Intriguingly, PrpRMt bound the *ramB* promoter region, which contains two imperfect palindromes, with an affinity (K_D_ = 32 nM) 10-times higher than that of the RamB protein (K_D_ = 328 nM). In contrast, the RamB protein did not interact with the *prpDC-prpR* promoter region, indicating different DNA-binding specificities for RamB and PrpR transcription factors, and suggesting that RamB might not play a role in regulating *prpDC* expression. Taken together, these results suggest that PrpRMt may not only be involved in the regulation of the methylcitrate cycle, but may also regulate the glyoxylate cycle by modulating the transcription of *prpDC*, *icl1*, and *ramB* genes.

In order to determine the role of PrpRMt in the transcriptional regulation of genes involved in fatty acid utilization during *M. tuberculosis* growth in media with different carbon sources, we investigated the expression levels of these genes (*prpDC*, *icl1*, and *ramB*) in wild-type and *prpR*-deletion strains. As expected, PrpRMt transcriptionally activated *prpDC* expression on medium containing products of odd-chain-length fatty acid catabolism (e.g., propionate), as evidenced by a ∼100-fold lower level of *prpDC* transcript in the Δ*prpR* mutant compared to the wild-type when grown in the presence of propionate as a sole carbon source (Figure 8A). This result is consistent with the impaired growth rate of the *M. tuberculosis* Δ*prpR* strain under these conditions (Figure 6C). Thus, the *prpR* gene appears to be indispensable for utilization of propionate and, as a consequence, propionyl-CoA, by tubercle bacilli. In recently published work, Griffin *et al.,* [27] obtained similar results; they did also demonstrate that growth on propionate requires the transcriptional induction of the propionyl-CoA-assimilating methylcitrate cycle enzymes, via the Rv1129c regulatory protein. Interestingly, a similar, but less-pronounced, change (∼50-fold) was also observed on 7H9+OADC rich medium (Figure 9A). In contrast, no substantial differences (i.e., ∼1.4-fold) were observed in *prpD* expression when the analyzed strains were cultivated on acetate (data not shown). Thus, our data suggest that PrpRMt-directed regulation of *prpDC* expression may be carbon-source-dependent. Moreover, similar to *prpDC*, *icl1* also exhibited PrpRMt-dependent regulation on propionate and rich 7H9+OADC broth (Figures 8B and 9B). Thus, these data, taken together with the direct interaction of PrpRMt with the *icl1* promoter, suggest the possible involvement of PrpRMt in the regulation of the glyoxylate cycle. However, *icl1* expression levels were not substantially different in Δ*prpR* and wild-type strains grown on acetate, in contrast to *icl1* levels in strains grown on propionate or rich medium. We also identified carbon source-dependent negative regulation of *ramB* expression by PrpRMt: in *M. tuberculosis* grown in a rich medium, PrpRMt weakly repressed *ramB* transcription; but in *M. tuberculosis* grown on propionate, this inhibitory effect was strengthened due to higher levels of *prpR* expression (Figure 8C and 9C). Taking into account the RamB-mediated *icl1* repression on glucose [11], we conclude that, under these conditions, the influence of PrpRMt on *icl1* transcription may be indirect. Nevertheless, these results suggest that PrpRMt plays a regulatory role in glyoxylate cycle control. Interestingly, Datta *et al*., [25] demonstrated that Rv1129 (PrpR) and Rv0455 (RamB) are required for the hypoxic response of their adjacent genes, *prpDC* and *icl1*, respectively.

It is worth mentioning that Micklinghoff *et al*. [19] suggested the involvement of an additional factor in derepression of *icl1* transcription during *M. tuberculosis* growth on acetate. However, since *prpR* expression on this carbon source is low and the product of this gene does not exert a dominant regulatory role, we postulate that PrpR does not act through interference with RamB binding to promote *icl1* expression. Thus, another regulator or additional regulatory mechanisms are presumably involved in controlling *icl1* expression levels under these conditions. However, the significantly higher affinity of PrpR for the *ramB* promoter compared to RamB suggests that when *M. tuberculosis* is grown on propionate or glucose, PrpRMt, acting directly and/or indirectly (i.e., through inhibition of *ramB* expression), is principally involved in regulating *icl1* expression.

Because the *icl1*-encoded isocitrate lyase is involved in both glyoxylate and methylcitrate cycles [15]–[17], the PrpRMt-dependent activation of *icl1* transcription on propionate may be directly associated with stringent control of *prpDC* expression under these conditions. It is well known that accumulation of propionate and methylcitrate cycle intermediates is toxic and has growth-inhibitory effects on bacteria [18], [28]. Thus, in the absence of ICL activity, *M. tuberculosis* cell growth on propionate is attenuated. However, we show here that, in the presence of propionate, PrpRMt acted as a transcriptional activator of the *prpDC* operon and the *icl1* gene, thus promoting the synthesis of enzymes necessary to metabolize this compound. Because PrpRMt also induced the expression of these genes on rich media (Fig. 9), we hypothesize that such regulation is mainly required to prevent the accumulation of toxic intermediates (e.g., propionate), a consideration that is particularly relevant during growth on fatty acids. This interpretation is consistent with the fact that *prpR* expression significantly increased in *M. tuberculosis* cells grown on propionate, thereby ensuring proper *prpDC* and *icl1* levels as well abolishing the inhibitory effect of RamB on *icl1* expression. As recently shown [29], *prpR* expression is also induced during the growth of *M. tuberculosis* on cholesterol as a sole carbon and energy source. These findings are in line with our results suggesting that PrpR is presumably involved in the regulation of genes responsible for controlling cholesterol utilization by the tubercle bacilli; PrpR binds strongly the promoter region of the *kstR* gene (Figure 10A and 10B) encoding the main regulator of the cholesterol catabolism [23], [24].

Surprisingly, *M. tuberculosis* lacking methylcitrate dehydratase and methylcitrate synthase (Δ*prpDC* mutant) replicates in mice similarly to the wild-type strain [16]. Munoz-Elias et al. postulated that *M. tuberculosis* uses the methylmalonyl-CoA pathway to metabolize propionyl-CoA for cell wall synthesis and maintenance [16]. They hypothesized that during infection of mice, *M. tuberculosis* presumably produces methyl-branched lipids in such large quantities that the pools of cytosolic propionyl-CoA are efficiently metabolized via the methylmalonyl-CoA pathway, thereby bypassing the requirement for the methylcitrate cycle [16]. However, further functional studies are required to clarify this hypothesis.

It is worth mentioning that *prpR* (*rv1129c*) orthologs are present in other pathogenic mycobacteria as well as in non-pathogenic *M. smegmatis* mc^2^155 strain (*msmeg_6643* locus), suggesting that the role of PrpR in the transcriptional regulation of genes involved in the metabolism of fatty acid β-oxidation products is conserved among *Mycobacterium* species.

Interestingly, *prpR* was identified as one of several genes whose expression is regulated by the σ^E^ factor [21], which has been shown to induce *prpR* expression in response to surface stress [21] and hypoxic conditions [25]. Our data clearly demonstrate the increased expression of *prpR* in the presence of propionate and the carbon source-dependent regulatory function of PrpR, suggesting additional complexities in the signals responsible for *prpR* activation and, as a consequence, regulation of gene expression by this transcription factor. Although further experiments are required to explain how carbon source regulates gene expression in *M. tuberculosis,* our results allow us to conclude that the role of PrpR protein in transcriptional regulation of crucial metabolic genes is not limited to a local *prpDC* target (Figure 11). Because it has been shown that *prpR* (*rv1129c*) is upregulated during *M. tuberculosis* persistence in macrophages and mice [10], we hypothesize that PrpRMt might play an important role in the adaptation of the pathogen to different conditions during infection of a human host.

## Materials and Methods

Ethics statement: Animal experiments were performed in strict accordance with the Polish regulations of the National Ethics Committee for Animal Experimentation and the European Health Law of the Federation of Laboratory Animal Science Associations (FELASA). The protocol to produce rabbit polyclonal antibodies raised against the PrpR protein was approved by the 1st Local Ethics Committee for Experiments with the Use of Laboratory Animals, Wroclaw, Poland (Permit Number: 3/2007, 17.01.2007), Institute of Immunology and Experimental Therapy, Polish Academy of Sciences, Wroclaw, Poland. All efforts were made to minimize suffering.

### DNA manipulations, bacterial strains, and culture conditions

DNA manipulations were carried out using standard protocols [30]. Enzymes were supplied by Fermentas and Promega; [γ-^32^P]ATP radioisotope was purchased from Hartmann Analytic; and oligonucleotides were synthesized by Genomed (Poland). The fidelity of all PCR-derived clones was confirmed by DNA sequencing. Bacterial strains, plasmids and oligonucleotides, as well as their relevant characteristic, are provided in Supplemental Material (Tables S3, S4, and S5). *Escherichia coli* culture conditions, media composition, antibiotic concentrations and transformation methods followed standard procedures [30]. The *M. tuberculosis* strain H37Rv and derivatives were cultured aerobically at 37°C in Middlebrook 7H9 broth or on 7H10 agar plates supplemented with 10% OADC (oleic acid-albumin-dextrose-catalase) enrichment, 0.05% Tween 80 (broth only) and 25 µg/ml kanamycin (when required). For growth rate analysis on defined carbon sources, *M. tuberculosis* strains were cultivated at 37°C for 7–14 days in M9 minimal salts medium [30] (without Tween-80) containing 2 mM MgSO_4_, 0.1 mM CaCl_2_ and glucose, sodium acetate or sodium propionate (0.5% each) as a carbon source. Growth of bacteria was monitored by measuring the OD at 600 nM (OD_600_) every 24 hours. For RNA extraction and gene expression measurements, strains were grown in 7H9+OADC broth (rich medium) or M9 minimal medium without Tween-80 containing either sodium acetate or sodium propionate (0.5%) as a sole carbon source.

### Expression and purification of 6His-tagged PrpRMt (Rv1129c) fusion protein

The *prpR* (*rv1129c*) gene of *M. tuberculosis* H37Rv was PCR-amplified from chromosomal DNA with primers Rv1129_Fw and Rv1129_Rv, and then cloned into the pET-28a(+) vector using *Bam*HI/*Xho*I restriction sites. The *M. tuberculosis* 6HisPrpR protein was expressed in the *E. coli* BL21 (DE3) strain transformed with the pET-28a(+)*prpRmt* vector. When cultures reached an OD_600_ of 0.9, recombinant 6HisPrpR protein synthesis was induced by addition of 0.1 mM IPTG (isopropyl-β-D-thio-galactoside), after which cells were incubated at 23°C overnight. Next, the bacterial culture was harvested and resuspended in buffer A (50 mM NaH_2_PO_4_, 300 mM NaCl, 10% glycerol) containing 20 mM imidazole (pH 7.3). After incubation on ice for 30 minutes in the presence of 1 mg/ml lysozyme, cells were disrupted by sonication. 6HisPrpRMt protein was purified by affinity chromatography using HIS-Select High Flow Nickel Affinity Gel according to the manufacturer's instructions (Sigma-Aldrich). The resin was washed with buffer A containing 20 mM imidazole until all contaminants had been removed, followed by an additional wash step with buffer A containing 40 mM imidazole. Recombinant 6HisPrpRMt protein was eluted from the resin with buffer B (50 mM NaH_2_PO_4_, 300 mM NaCl, 10% glycerol, 250 mM imidazole, pH 7.3). Sodium dodecyl sulfate-polyacrylamide gel electrophoresis (SDS-PAGE) analysis [31] of elution fractions showed that the recovered fusion protein was 95% pure.

### EMSA analysis of 6HisPrpRMt-promoter binding

Interactions of recombinant 6HisPrpRMt protein with DNA fragments containing *prpDR* (*rv1130-rv1129c*), *icl1* (*rv0467*), *ramB* (*rv0465c*) and *kstR* (*rv3574*) promoter regions were investigated by EMSA. The respective DNA fragments were amplified by PCR with specific primers (Table S5) and eluted from agarose gels using QIAquick Gel Extraction Kits (Qiagen). A DNA fragment that does not bind 6HisPrpRMt (negative control) was also prepared in the same manner. DNA probe (100–150 ng) was incubated at 25°C for 30 minutes with increasing amounts of purified 6HisPrpRMt protein, appropriately diluted in 1x Binding Buffer (50 mM Tris-HCl pH 7.5, 50 mM KCl, 10 mM MgCl_2_, 10% glycerol, 0.5 mM EDTA). The resulting nucleoprotein complexes were resolved on 4% polyacrylamide gels in 0.25x TBE (Tris-borate-acetate) buffer at 5–10 V/cm. The complexes were analyzed using a Typhoon 8600 Variable Mode Imager and ImageQuant 5.2 software (Molecular Dynamics).

### SPR analysis of 6HisPrpRMt-promoter binding

Protein-DNA interactions were analyzed in real time using SPR. DNA fragments containing *prpDR*, *ramB* and *kstR* promoter regions were generated by PCR using specific primers, one of which was biotin-labeled (Table S5), and then immobilized on the streptavidin-coated SA Sensor Chip of a BIAcore T3000 device (GE Healthcare). An additional DNA fragment not recognized by 6HisPrpRMt served as a negative control. Approximately 100 response units (RUs) of each DNA were immobilized. DNA loosely attached to the chip surface was removed by applying 0.1% SDS (5 seconds at a flow rate of 15 µl/min). In order to exclude the effects of mass transport on the kinetics of protein-DNA interactions, we performed measurements at various 6HisPrpRMt concentrations (0.1–1 µM) at a continuous flow rate (15 µl/min). Protein interactions were analyzed in 1x Binding Buffer. At the end of each cycle, bound 6HisPrpRMt protein was removed by washing with 0.1% SDS (5 seconds at 15 µl/min). The results were plotted as sensograms after subtraction of the background response signal obtained in a negative control experiment. BIAevaluation Software 4.1 (GE Healthcare) was used for data analysis.

### DNase I footprinting

For DNase I footprinting experiments, 260- and 250 bp PCR products encompassing *prpDR* and *ramB* promoters, respectively, were amplified with specific primers (Table S5), one of which was previously 5′ radiolabeled. The respective DNA probes were agarose-purified and used directly in protein-interaction assays. Various amounts of 6HisPrpRMt protein in 1x Binding Buffer were incubated with radiolabeled DNA fragments (10 fmol) at 25°C for 30 minutes. Subsequently, probes were treated with DNase I (0.01 U/µl) at 37°C for 5 minutes, and cleavage products were separated on 8% polyacrylamide-urea sequencing gels. Gels were analyzed using a Typhoon 8600 Variable Mode Imager and ImageQuant 5.2 software (Molecular Dynamics).

### Gene replacement construct and disruption of the *M. tuberculosis prpR* gene

To perform unmarked deletion of the *prpR* (*rv1129c*) *M. tuberculosis* gene, we used a suicidal recombination delivery vector based on p2NIL [32]. The recombination vector carried the 5′ *prpR* upstream region (1,604 bp) and the first 198 bp of the *prpR* gene tagged to the 3′ part of the *prpR* gene (212 bp), followed by 1457 bp of the *prpR* downstream region. PCR products containing 5′ and 3′ fragments of the gene were ligated into *Sca*I/*Pac*I restriction sites of the p2NIL vector; the resultant Δ*prpRmt* gene encoded a non-functional protein lacking the HTH motif. The final vector, p2NILΔ*prpRmt*_OK, also included the screening *Pac*I cassette from pGOAL17 [32]. A gene replacement strategy was used to disrupt *prpRmt* at its native locus on the chromosome. The plasmid DNA was treated with UV light (100 mJ/cm^2^) and integrated into *M. tuberculosis* chromosome by homologous recombination. The resulting single-crossover mutant colonies were blue, kanamycin resistant, and sensitive to sucrose. The recombination site was confirmed by PCR and Southern blot hybridization. The single-crossover strains were further processed to select for double-crossover mutants, which were white, kanamycin-sensitive, and resistant to sucrose (2%). Wild-type and double-crossover mutants were distinguished using PCR and Southern blotting (Figure S3 and S4). Complementation of the *M. tuberculosis* Δ*prpR* strain was performed by introducing a functional copy of the *prpRmt* gene, cloned into pMV306 integration vector together with 5′ *prpRmt* upstream region (300 bp; likely containing its own promoter). Incorporation of additional nucleotides within the complementation construct during DNA cloning was avoided by exploiting a *Cla*I restriction site located within the *prpRmt* gene (622–627 bp). A *Hin*dIII/*Cla*I-flanked DNA fragment containing the 5′ *prpRmt* upstream region (300 bp) and first 627 bp of the *prpRmt* gene was PCR-amplified with specific primers (Table S5), whereas the second part of *prpRmt* (628–1461 bp) was excised directly from the pUT18C*prpRmt* vector using *Cla*I/*Kpn*I restriction enzymes. Next, both parts of the *prpRmt* gene were ligated into *Hin*dIII/*Kpn*I sites of the pMV306 vector. Complementation of the unmarked deletion of the *prpR* wild-type gene was achieved by electrotransformation of the final pMV306*prpRmt* construct into *M. tuberculosis* Δ*prpR* and integration into the *attB* site of the genome, yielding the *M. tuberculosis* Δ*prpR*+pMV*prpR* strain.

### Preparation of PrpRMt antibodies

Anti-PrpRMt antisera were obtained by immunization of rabbits with purified *M. tuberculosis* 6HisPrpR protein mixed with TiterMax Gold Adjuvant, according to the manufacturer's instructions (Sigma). An additional booster injection was given after 2 weeks. Serum samples were collected 2 weeks after the booster injection. Cells and cellular debris were removed by centrifugation (1000 g, 30 minutes, 4°C) and the serum was stored at −20°C. IgG was purified by ammonium sulfate precipitation (to 40% saturation) and dialysis against 5 mM Tris-HCl (pH 8.0), followed by affinity chromatography. The affinity column was prepared by immobilization of 4 mg purified recombinant PrpRMt protein on CNBr-activated Sepharose, according to the manufacturer's recommendations (Sigma). Antibodies in phosphate-buffered saline (PBS; pH 7.3) were applied to the affinity column, washed with PBS, eluted with 0.1 M glycine (pH 2.5), and neutralized with 1 M Tris-HCl (pH 9.5) buffer. The specificity of collected antibody samples was analyzed using standard alkaline phosphate activity-based Western blotting.

### Whole-cell immunoprecipitation assay

*M. tuberculosis* H37Rv wild-type and *prpR-*deletion strains cultivated in 7H9+OADC broth at 37°C to an OD_600_ of 0.9 were cross-linked with glutaraldehyde (1%) for 5 min, as described previously [33]. Cells not subjected to cross-linking and mutant strain cells, treated in the same manner as experimental samples, served as negative controls. Subsequent steps of the immunoprecipitation assay were carried out according to a previously described protocol [34]. Immunoprecipitated DNA released from protein complexes was phenol extracted, ethanol precipitated, and resuspended in TE buffer [30]. Precipitated DNA samples were analyzed by semiquantitative PCR (25 cycles) using three primer sets (Table S5) that amplified ∼250-bp DNA fragments encompassing *prpDR* and *icl1* promoter regions, and a negative control region. Input DNA (not immunoprecipitated) subjected to the same cross-link reversal and purification procedures as the experimental samples served as a positive control for PCR. Following electrophoresis, the gel was analyzed using a Typhoon 8600 Variable Mode Imager and ImageQuant 5.2 software (Molecular Dynamics).

### RNA extraction and reverse transcription

RNA was extracted from *M. tuberculosis* cultures incubated in 7H9+OADC broth and M9 minimal medium containing either acetate or propionate as a carbon source. Since this minimal medium does not sustain robust growth of *M. tuberculosis*, bacteria were precultured in 7H9+OADC broth to an OD_600_ of 0.6-0.8. Next, cells were washed twice with M9 minimal medium containing acetate or propionate, resuspended in the respective minimal medium (without Tween-80), and incubated at 37°C for an additional 48 hours. The cultures were then collected by centrifugation (6000 g, 10 minutes, 4°C), and bacterial pellets were resuspended in the TRIzol reagent (Invitrogen). The cells were then disrupted in the presence of silica beads (BioSpec Products) using a Mini-BeadBeater-8 (BioSpec Products) for 3 minutes. Subsequent steps of RNA purification were performed according to manufacturer's protocol (Invitrogen) with minor modifications. The tubes were centrifuged (16000 g, 15 minutes, 4°C), and the supernatants were mixed with chloroform. Each sample was recentrifuged using the same conditions, and the upper phase was collected and mixed with an equal volume of isopropanol. After 60 minutes incubation at room temperature, the samples were centrifuged (16000 g, 30 min, 4°C). The precipitated RNA pellet was washed with ethanol (75%), air-dried, and resuspended in water. Total RNA was subsequently treated with DNase I (Amplification Grade) according to the manufacturer's instruction (Invitrogen) and directly used in reverse transcription reactions. RNA concentration and purity were determined spectrophotometrically and by agarose gel electrophoresis.

Before reverse transcription, DNase I-digested RNA samples were used as templates in standard PCR to verify efficient nuclease treatment. The reverse transcription reactions were carried out using a SuperScript III First-Strand Synthesis SuperMix kit and random hexanucleotides, according to the manufacturer's protocol (Invitrogen). The reaction products were analyzed by agarose gel electrophoresis.

### Quantitative real-time PCR

SYBR green-based real-time PCR (2x HS-PCR Mix SYBR A; A&A Biotechnology) was used for the quantification of mRNA levels of *prpR*, *prpDC*, *icl1*, and *ramB* genes in *M. tuberculosis* H37Rv wild-type, Δ*prpR*, and complemented strains cultivated on rich 7H9+OADC medium or M9 minimal medium with acetate or propionate as a carbon source. To exclude the possibility of secondary structure formation, we used the Primer Express 3.0 application to design primers (Table S5). *prpRmt* expression in all tested *M. tuberculosis* strains grown on 7H9+OADC broth was measured using primers specific to the deleted portion of the gene (Figure S5). Reactions were performed on a StepOne Plus apparatus with StepOne Software 2.0 application, according to the manufacturer's instructions (Applied Biosystems). The relative quantity of target gene mRNA was determined by reference to the mRNA levels of the *M. tuberculosis* housekeeping gene, *sigA*. Three independent experiments with cDNA templates derived from RNA isolated from three independent *M. tuberculosis* strain cultures were carried out.

### Statistical Analysis

The difference between different experimental groups was determined by Student's t-test. P-values of <0.05 were considered significant. P-values were calculated using Excel 2007 software (Microsoft Office).

## Supporting Information

Figure S1 **PrpRMt protein oligomerization assay.** A. 6HisPrpRMt protein (5 µg, lane 1) was incubated in the presence of increasing concentrations of glutaraldehyde (2.5 mM, lane 2; 5 mM, lane 3; and 10 mM, lane 4). Cross-linked protein was analyzed by SDS-PAGE on 10% gels. M, Unstained Protein Molecular Weight Marker (Fermentas). B. Analysis of PrpRMt dimerization in a bacterial two-hybrid system. The *E. coli* BTH101 strain was co-transformed with pUT18C*prpRmt* and pKT25*prpRmt* plasmids carrying the *prpRmt* gene fused to the T18 domain and the T25 domain of *cyaA* from *B. pertussis*, respectively [35].
(TIF)

Figure S2 **Analysis of 6HisRamB protein binding to the** ***prpDR*** **promoter region by EMSA.** Oligonucleotides corresponding to the promoter regions of (A) *prpDR* (260 bp) or (B) *mtrA* (251 bp; negative control) were incubated with increasing amounts of 6HisRamB protein; protein-DNA mixtures were analyzed on 4% polyacrylamide gels.
(TIF)

Figure S3 **PCR-based analysis of** ***prpRmt*** **gene deletion.** Agarose gel electrophoresis of PCR products obtained from chromosomal DNA isolated from the *M. tuberculosis prpR-*deletion mutant (lanes 1 and 2) and wild-type strain (lanes 3 and 4). Rv1129_Fw and Rv1129_Rv primers (Table S5) encompassing the *prpRmt* gene were used in each reaction. M, GeneRuler 1 kb DNA Ladder (Fermentas).
(TIF)

Figure S4 **Southern blot analysis of** ***prpRmt*** **gene deletion in the** ***M. tuberculosis*** **chromosome.** Chromosomal DNA isolated from the *M. tuberculosis prpR*-deletion mutant (lanes 1–5), wild-type strain (lanes 6, 7), and single cross-over *prpR* mutant (lanes 8, 9) were digested with *Sal*I restriction enzyme, yielding 556 bp (mutated allele) or 1607 bp (wild type) DNA fragments. Single cross-over recombinants contained both alleles. The p2NILΔ*prpRmt*_1+2 vector (Table S4) was used as a hybridization probe. Analyses revealed that the putative *prpRmt* deletion strain (lane 2) was a single cross-over mutant. M, GeneRuler 1 kb DNA Ladder (Fermentas).
(TIF)

Figure S5 ***prpRmt*** **expression levels in tested** ***M. tuberculosis*** **strains grown on 7H9+OADC broth.** qPCR analysis of *prpR* expression in *M. tuberculosis* H37Rv (wild-type), Δ*prpR*, and Δ*prpR*+pMV*prpR* (complemented) strains cultivated on rich 7H9+OADC medium. *prpR* expression levels were normalized to those in the wild-type strain (set to 1). Means were calculated from three independent experiments and three determinations per experiment. Error bars represent standard errors of the mean. Statistical significance was calculated by the Student's t-test.
(TIF)

Figure S6 **PCR-based analysis of the** ***prpRmt*** **transcription start site.** Agarose gel electrophoresis of PCR products obtained from cDNA template derived from the *M. tuberculosis* Δ*prpR*+pMV*prpR* strain cultivated on propionate as the sole carbon source. In each reaction, primer p1129map_Rv was used together with the following forward primers (Table S5): p1129map_Fw: 1 (lane 1), 2 (lane 2), 3 (lanes 3) (panel A); 3Up1 (lanes 4), 3Up2 (lane 5), 3Up3 (lane 6), 3Down1 (lane 7) (panel B); 2Fw3Up1 (lane 8), 2Up1 (lane 9) (panel C). Lanes C1-C9 contain PCR products obtained on the chromosomal DNA as a template (positive control) with the same pair of primers. M – λ DNA digested with the *Pst*I restriction enzyme. Panel D shows schematic representation of the experiment and binding sites for particular pair of primers used in the analysis. Increasing numbers of particular map_Fw primer (Table S5) correspond with the numbers of lanes in the agarose gel electrophoresis of obtained PCR products.
(TIF)

Table S1 **Location of potential PrpRMt targets within the** ***M. tuberculosis*** **H37Rv chromosome.**
(RTF)

Table S2 **Comparison of 8-meric sequences located within PrpRMt-recognized promoter regions.**
(RTF)

Table S3 **Bacterial strains used in this study.**
(RTF)

Table S4 **Plasmids used in this study.**
(RTF)

Table S5 **Oligonucleotides (primers) used in DNA cloning and qPCR.**
(DOC)
