# Correction: A Novel Role of the PrpR as a Transcription Factor Involved in the Regulation of Methylcitrate Pathway in *Mycobacterium tuberculosis*

**DOI:** 10.1371/journal.pone.0113015

**Published:** 2014-11-07

**Authors:** The PLOS ONE Staff

There are errors in the Funding section. The correct funding information is as follows: The research was supported by Wroclaw Research Centre EIT+ under the project “Biotechnologies and advanced medical technologies” – BioMed (POIG.01.01.02-02-003/08) financed from the European Regional Development Fund (Operational Programme Innovative Economy, 1.1.2). JZC and PM gratefully acknowledge financial support received from the Foundation for Polish Science (MISTRZ Programme) (http://www.fnp.org.pl/index.php?lng=en). The funders had no role in study design, data collection and analysis, decision to publish, or preparation of the manuscript.
