# Correction: A Novel Role of the PrpR as a Transcription Factor Involved in the Regulation of Methylcitrate Pathway in *Mycobacterium tuberculosis*

**DOI:** 10.1371/journal.pone.0208565

**Published:** 2018-12-03

**Authors:** Paweł Masiewicz, Anna Brzostek, Marcin Wolański, Jarosław Dziadek, Jolanta Zakrzewska-Czerwińska

An incorrect image is used in Fig 4B p*icl1* panel of this article [1], which is duplicated in Fig 2B p*dnaA* panel of an article published in *Antonie Van Leeuwenhoek* [2].

**Fig 4:**
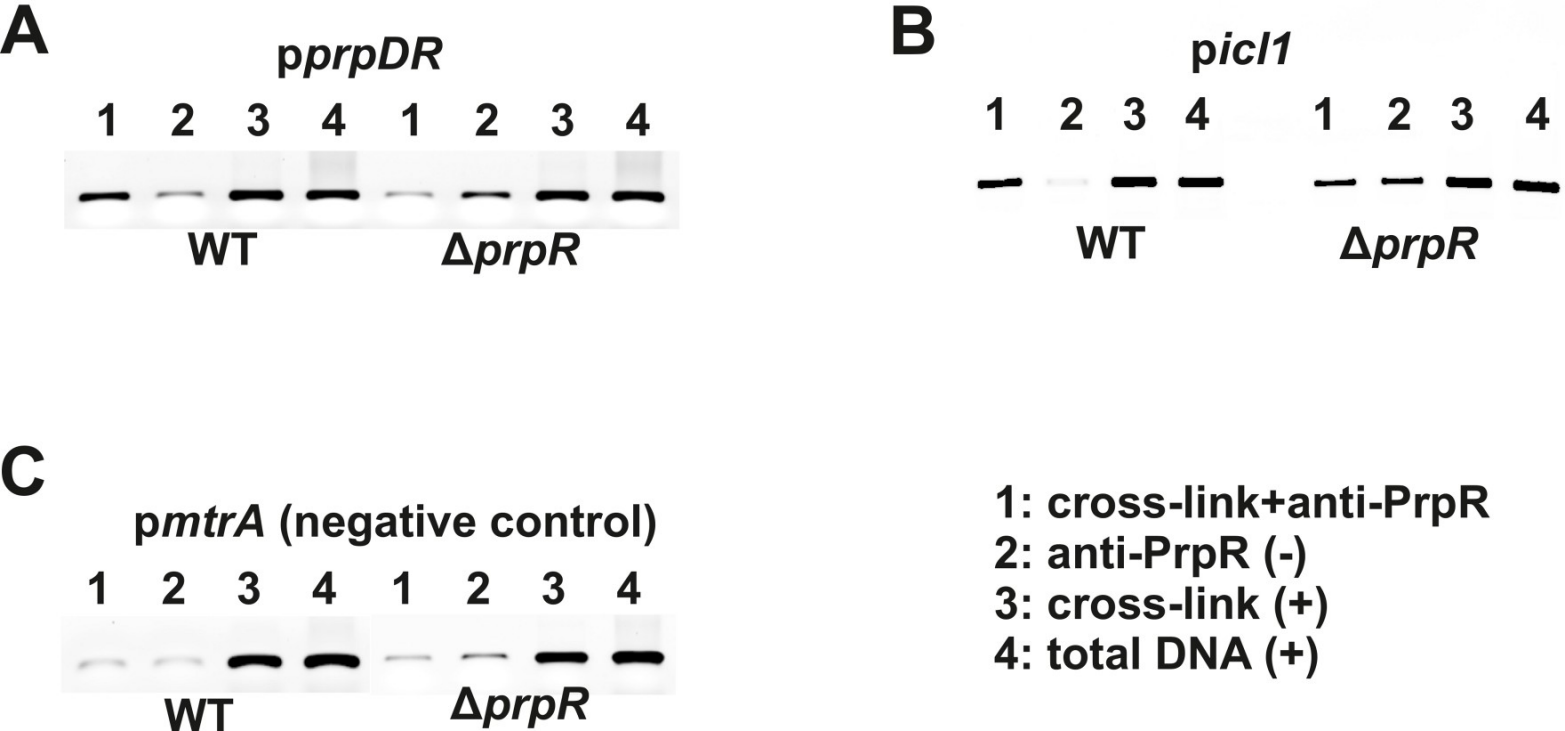
PrpR binds the promoter region of *prpDC* and *icl1* genes in intact *M*. *tuberculosis* cells. Identification of intracellular PrpR-DNA complex using immunoprecipitation. PrpR-DNA complexes cross-linked with glutaraldehyde were immunoprecipitated with anti-6HisPrpRMt polyclonal antibodies (sample 1). PCR was carried out with the primer pairs, p1129_Fw and p1129_Rv (*pprpDR*)(A); picl_Fw and picl_Rv (*picl1*)(B); and pmtrA_Fw and pmtrA_Rv (*pmtrA*, negative control)(C). Negative control (2) consisted of DNA template extracted from the cells subjected to immunoprecipitation, but nucleoprotein complexes were not previously cross-linked. Positives controls (+) were also performed using template obtained from strains subjected only to cross-linking (3) or total DNA extracted from the cells (4).

The authors apologize for this error, and clarify that the panel in question represents the p*dnaA* experimental results, as reported in [2].

The authors provide an updated Fig 4 which includes the correct image for p*icl1*, and confirm that the image represents the experiment carried out at the same time as other images in the figure. The authors also provide raw data underlying Fig 4 as Supporting Information files.

Moreover, the authors reused Fig 2A from the *PLOS ONE* article [1] in the *Antonie Van Leeuwenhoek* article [2] as part of Fig 2A without attribution of the original publication. Additionally, the authors reused Fig 4A and 4C from the *PLOS ONE* article [1] in the *Antonie Van Leeuwenhoek* article [2] as part of Fig 2B without attribution of the original publication. The authors apologize for this oversight which has been corrected [3]. The authors would like to mention that the data duplicated in the *Antonie Van Leeuwenhoek* article [2] represent the positive and the negative controls in the corresponding experiments and were reused unintentionally only for easier data comparison.

## Supporting information

S7 Fig The immunoprecipitation data used in Fig 4, specifically, the upper row (lanes 2–9) contains raw data used in panel A (Fig 4A), the bottom row (lanes 11–14) contains raw data used in Δ*prpR* part of panel 4C (Fig 4C). Note that the data shown in the upper row (lanes 11–18) correspond to the data that were originally and mistakenly used in the Fig 4B of the *PLOS ONE* article as p*icl1* panel, these lanes represent experimental results for the p*dnaA* panel as reported in [2] (this issue is referred in the current erratum).
(TIF)

S8 Fig The immunoprecipitation data used in the corrected Fig 4, specifically the upper row (lanes 2–5 and 7–10) contains raw data used in Fig 4B, p*icl1* panel. (TIF)

S9 Fig The immunoprecipitation data used in Fig 4, specifically, the bottom row (lanes 11–14) contains raw data used in WT part of panel 4C (Fig 4C). (TIF)
