# Supplementary figures and images for: A Novel Role of the PrpR as a Transcription Factor Involved in the Regulation of Methylcitrate Pathway in Mycobacterium tuberculosis
Source: PLoS One. 2012 Aug 16;7(8):e43651. doi: 10.1371/journal.pone.0043651 (PMC3420887; doi:10.1371/journal.pone.0043651)

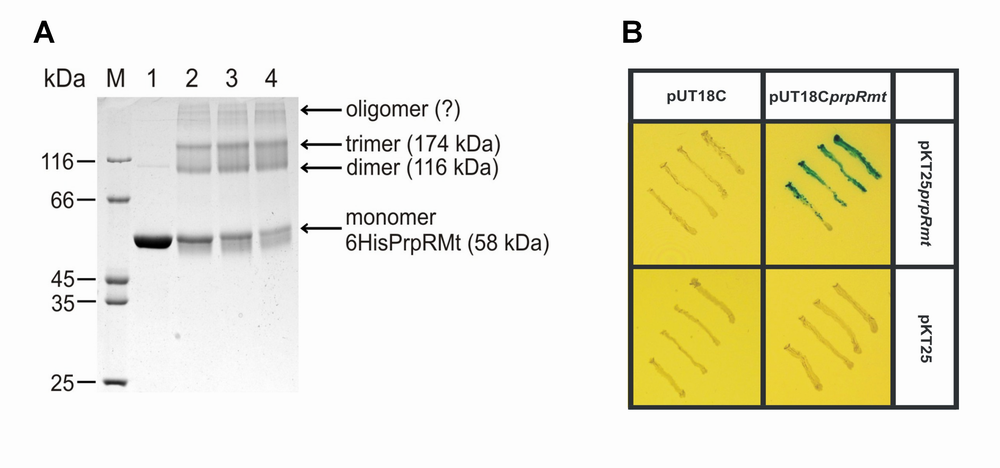

Supplement: Figure S1 — PrpRMt protein oligomerization assay. A. 6HisPrpRMt protein (5 µg, lane 1) was incubated in the presence of increasing concentrations of glutaraldehyde (2.5 mM, lane 2; 5 mM, lane 3; and 10 mM, lane 4). Cross-linked protein was analyzed by SDS-PAGE on 10% gels. M, Unstained Protein Molecular Weight Marker (Fermentas). B. Analysis of PrpRMt dimerization in a bacterial two-hybrid system. The E. coli BTH101 strain was co-transformed with pUT18CprpRmt and pKT25prpRmt plasmids carrying the prpRmt gene fused to the T18 domain and the T25 domain of cyaA from B. pertussis, respectively [35]. (TIF) [file pone.0043651.s001.tif]

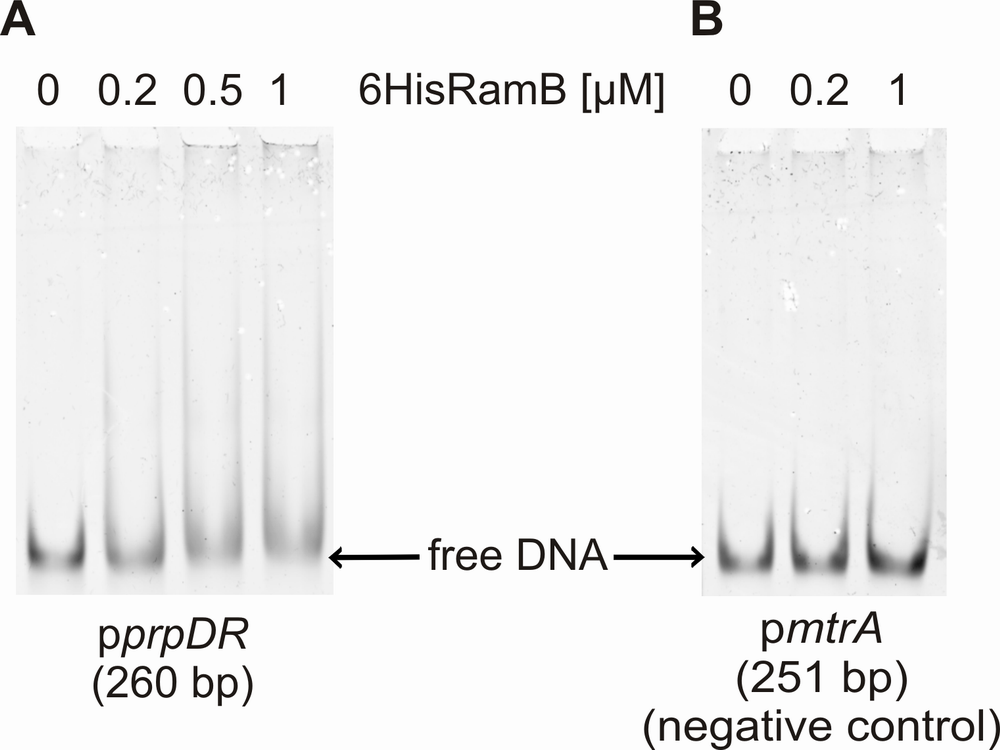

Supplement: Figure S2 — Analysis of 6HisRamB protein binding to the prpDR promoter region by EMSA. Oligonucleotides corresponding to the promoter regions of (A) prpDR (260 bp) or (B) mtrA (251 bp; negative control) were incubated with increasing amounts of 6HisRamB protein; protein-DNA mixtures were analyzed on 4% polyacrylamide gels. (TIF) [file pone.0043651.s002.tif]

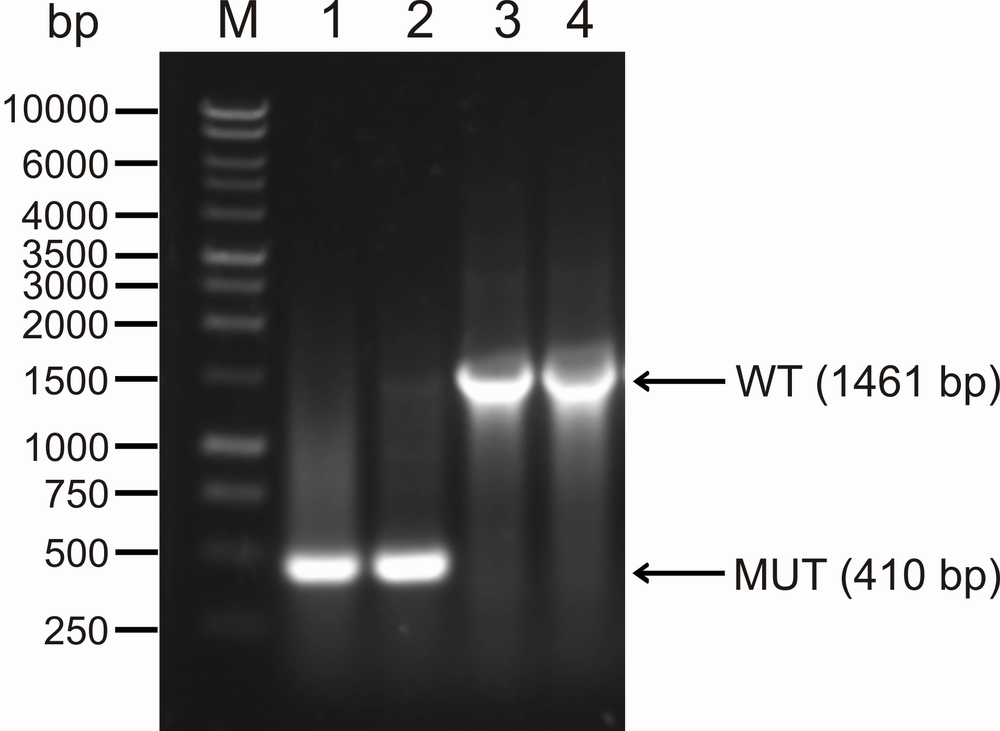

Supplement: Figure S3 — PCR-based analysis of prpRmt gene deletion. Agarose gel electrophoresis of PCR products obtained from chromosomal DNA isolated from the M. tuberculosis prpR-deletion mutant (lanes 1 and 2) and wild-type strain (lanes 3 and 4). Rv1129_Fw and Rv1129_Rv primers (Table S5) encompassing the prpRmt gene were used in each reaction. M, GeneRuler 1 kb DNA Ladder (Fermentas). (TIF) [file pone.0043651.s003.tif]

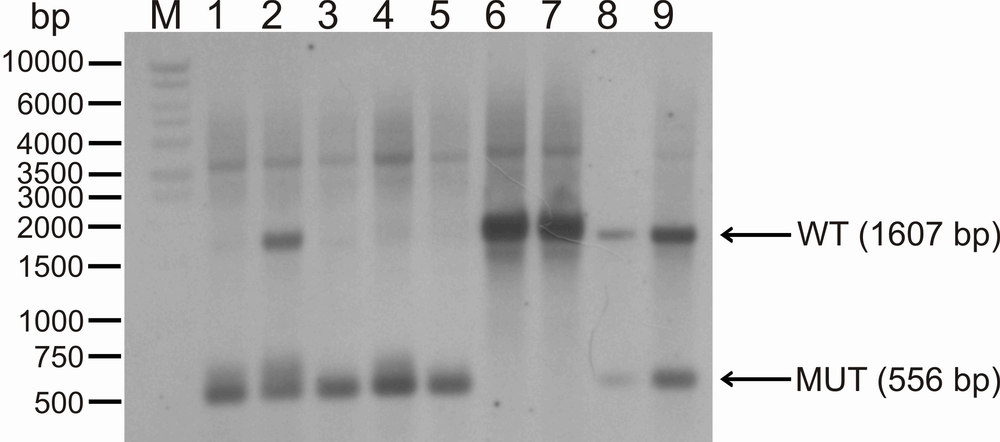

Supplement: Figure S4 — Southern blot analysis of prpRmt gene deletion in the M. tuberculosis chromosome. Chromosomal DNA isolated from the M. tuberculosis prpR-deletion mutant (lanes 1–5), wild-type strain (lanes 6, 7), and single cross-over prpR mutant (lanes 8, 9) were digested with SalI restriction enzyme, yielding 556 bp (mutated allele) or 1607 bp (wild type) DNA fragments. Single cross-over recombinants contained both alleles. The p2NILΔprpRmt_1+2 vector (Table S4) was used as a hybridization probe. Analyses revealed that the putative prpRmt deletion strain (lane 2) was a single cross-over mutant. M, GeneRuler 1 kb DNA Ladder (Fermentas). (TIF) [file pone.0043651.s004.tif]

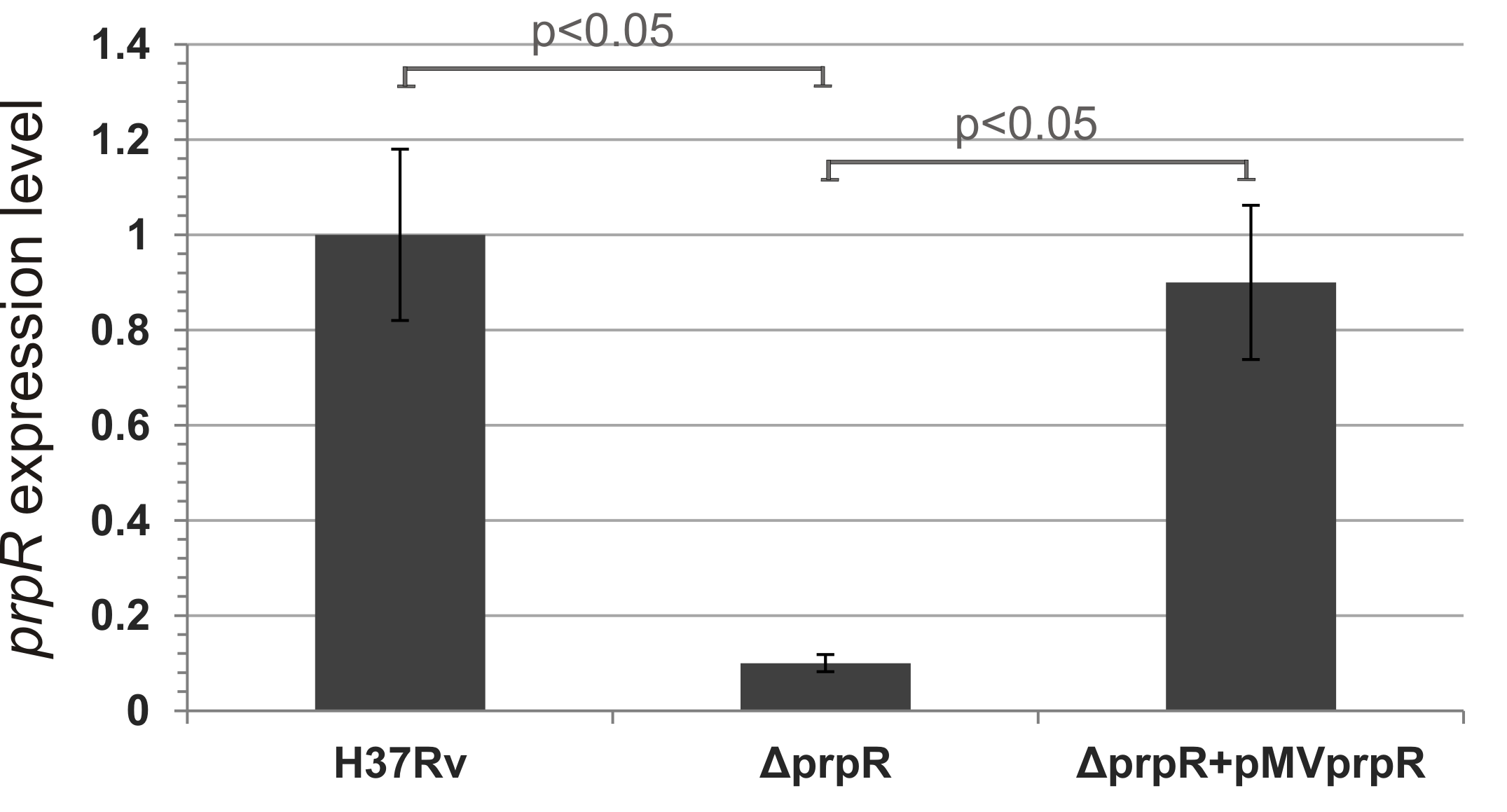

Supplement: Figure S5 — prpRmt expression levels in tested M. tuberculosis strains grown on 7H9+OADC broth. qPCR analysis of prpR expression in M. tuberculosis H37Rv (wild-type), ΔprpR, and ΔprpR+pMVprpR (complemented) strains cultivated on rich 7H9+OADC medium. prpR expression levels were normalized to those in the wild-type strain (set to 1). Means were calculated from three independent experiments and three determinations per experiment. Error bars represent standard errors of the mean. Statistical significance was calculated by the Student's t-test. (TIF) [file pone.0043651.s005.tif]

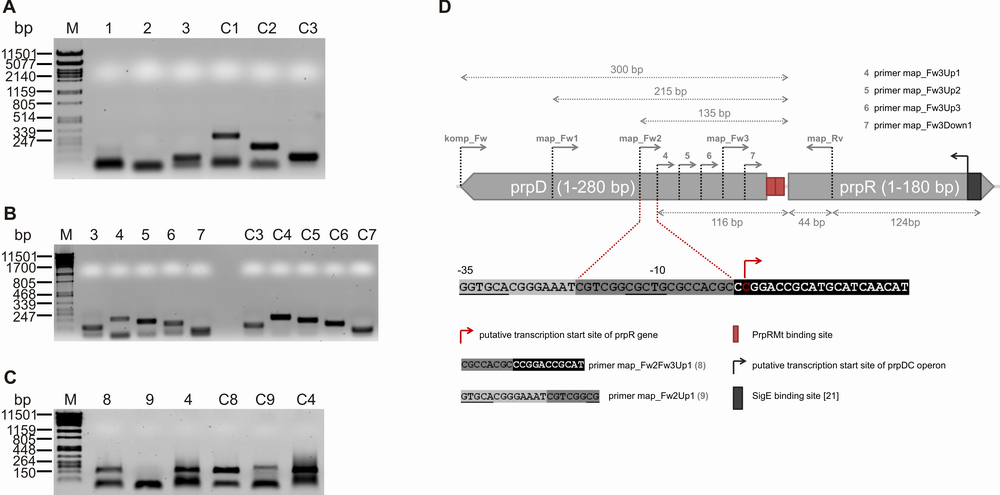

Supplement: Figure S6 — PCR-based analysis of the prpRmt transcription start site. Agarose gel electrophoresis of PCR products obtained from cDNA template derived from the M. tuberculosis ΔprpR+pMVprpR strain cultivated on propionate as the sole carbon source. In each reaction, primer p1129map_Rv was used together with the following forward primers (Table S5): p1129map_Fw: 1 (lane 1), 2 (lane 2), 3 (lanes 3) (panel A); 3Up1 (lanes 4), 3Up2 (lane 5), 3Up3 (lane 6), 3Down1 (lane 7) (panel B); 2Fw3Up1 (lane 8), 2Up1 (lane 9) (panel C). Lanes C1-C9 contain PCR products obtained on the chromosomal DNA as a template (positive control) with the same pair of primers. M – λ DNA digested with the PstI restriction enzyme. Panel D shows schematic representation of the experiment and binding sites for particular pair of primers used in the analysis. Increasing numbers of particular map_Fw primer (Table S5) correspond with the numbers of lanes in the agarose gel electrophoresis of obtained PCR products. (TIF) [file pone.0043651.s006.tif]
