# Supplementary material for: A Novel Role of the PrpR as a Transcription Factor Involved in the Regulation of Methylcitrate Pathway in Mycobacterium tuberculosis
Source: PLoS One. 2012 Aug 16;7(8):e43651. doi: 10.1371/journal.pone.0043651 (PMC3420887; doi:10.1371/journal.pone.0043651)
Supplement: Table S1 — Location of potential PrpRMt targets within the M. tuberculosis H37Rv chromosome. (RTF) [file pone.0043651.s007.rtf]

Table S1.	Locations of potential PrpRMt targets within the M. tuberculosis H37Rv chromosome.

No.	Location within chromosome 
[in bp]*	Sequence	Gene name or promoter region (chromosome locus) and location of the sequence relative to the start codon	
1	371095	TTTGCAAA	ppe5 (rv0304c) gene (+1663 bp)	
2	375011	TTTGCAAA	ppe6 (rv0305c) gene (+694 bp)	
3	393331	TTTGCAAA	cyp135A1 (rv0327c) gene (+708 bp)	
4	557352	TTTGCAAA	icl1 (rv0467) promoter region (-175 bp) 	
5	1056968	TTTGCAAA	pgi (rv0946c) promoter region (-290 pz)	
6	1254545	TTTGCAAA	common prpR (rv1129c) (-18 bp) and prpDC (rv1130-1131) promoter region (-10 bp)	
7	1865555	TTTGCAAA	argC (rv1652) promoter region (-21 bp) 	
8	2164706	TTTGCAAA	ppe34 (rv1917c) gene (+2599 bp)	
9	2637842	TTTGCAAA	ppe40 (rv2356c) gene (+1687 bp)	
10	2934433	TTTGCAAA	pdxH (rv2607) gene (+236 bp)	
11	3667171	TTTGCAAA	accA3 (rv3285) gene (+815 bp)	
12	4016382	TTTGCAAA	kstR (rv3574) promoter region (-102 bp)	
13	4213973	TTTGCAAA	rv3767c promoter region (-40 bp)	
14	4359662	TTTGCAAA	espK (rv3879c) gene (+114 bp)	
15	4404702	TTTGCAAA	rv3916c gene (+459 bp)	
*Asterisk indicates first nucleotide of the sequence.

The M. tuberculosis H37Rv genome sequence (4,411,532 bp in length) was searched in silico using the Fuzznuc application (http://anabench.bcm.umontreal.ca/anabench/Anabench-Jsp/Applications/fuzznuc.jsp) with the TTTGCAAA sequence as input.  
