# Supplementary material for: A Novel Role of the PrpR as a Transcription Factor Involved in the Regulation of Methylcitrate Pathway in Mycobacterium tuberculosis
Source: PLoS One. 2012 Aug 16;7(8):e43651. doi: 10.1371/journal.pone.0043651 (PMC3420887; doi:10.1371/journal.pone.0043651)
Supplement: Table S2 — Comparison of 8-meric sequences located within PrpRMt-recognized promoter regions. (RTF) [file pone.0043651.s008.rtf]

Table S2.	Comparison of 8-meric sequences located within PrpRMt-recognized promoter regions.
No.	Sequence (5'-3')	Promoter region	Estimated PrpRMt affinity*	
1	TTTGCAAA	prpDR	++++	
2	TTTGCGAA			
3	TTTGCAAA	icl1	++++	
4	TTTGCGAA			
5	TTTGCAAA	argC	++++	
6	TGTGCATA			
7	TTTGCAAA	kstR	+++	
8	TTTGCAAA	rv3767c	+++	
9	CTTGCTAA	ramB	++	
10	TCTGCGAA			
Bold font denotes nucleotides consistent with a perfect 8-bp palindrome.
*Estimations of PrpRMt affinity to analyzed promoter regions were determined based on EMSA results.
All six promoter regions containing PrpRMt-recognized sequence were tested in EMSA experiments. The migration of each DNA fragment encompassing the analyzed promoter was specifically retarded by increasing amounts of PrpRMt protein (some data not shown). 
