# Supplementary material for: A Novel Role of the PrpR as a Transcription Factor Involved in the Regulation of Methylcitrate Pathway in Mycobacterium tuberculosis
Source: PLoS One. 2012 Aug 16;7(8):e43651. doi: 10.1371/journal.pone.0043651 (PMC3420887; doi:10.1371/journal.pone.0043651)
Supplement: Table S3 — Bacterial strains used in this study. (RTF) [file pone.0043651.s009.rtf]

Table S3.	Bacterial strains used in this study.

Strain	Genotype	Source	
Escherichia coli	
DH5	F-, 80dlacZM15, recA1, endA1, gyrA96, thi-1, hsdR17, (rk-, mk+), supE44, relA1, deoR, (lacZYA-argF)U169	Laboratory stock	
BL21 (DE3)	F-, ompT, hsdSB(rB-, mB-), dcm, gal, ë(DE3)	Stratagene	
BTH101	F-, cya-99, araD139, galE15, galK16, rpsL1 (strR), hsdR2, mcrA1, mcrB1	[34]	
WM1905	F-, dam-13::Tn9 (camR), dcm-6, hsdR2 (rk-, mk+), leuB6, hisG4, thi-1, araC14, lacY1, galK2, galT22, xylA5, mtl-1, rpsL136 (strR), fhuA31, tsx-78, glnV44, mcrA, mcrB1	[35]	
Mycobacterium	
M. tuberculosis H37Rv	Laboratory strain [2]	Laboratory stock	
M. tuberculosis ÄprpR	M. tuberculosis H37Rv with deleted prpRmt gene (unmarked deletion)	This work	
M. tuberculosis ÄprpR+pMVprpR	M. tuberculosis ÄprpR complemented strain containing a pMV306-integrated functional copy of the prpRmt gene under control of its own promoter  	This work	
