# Supplementary material for: A Novel Role of the PrpR as a Transcription Factor Involved in the Regulation of Methylcitrate Pathway in Mycobacterium tuberculosis
Source: PLoS One. 2012 Aug 16;7(8):e43651. doi: 10.1371/journal.pone.0043651 (PMC3420887; doi:10.1371/journal.pone.0043651)
Supplement: Table S4 — Plasmids used in this study. (RTF) [file pone.0043651.s010.rtf]

Table S4.	Plasmids used in this study.

Plasmid	Relevant characteristics	Source	
pGEM-T Easy	T/A cloning vector; ampR	Promega 	
pET-28a(+)	Expression vector; kanR	Novagen	
pET-28a(+)prpRmt	pET-28a(+) derivative carrying prpRmt (rv1129c) gene; kanR	This work 	
pET-28a(+)ramB	pET-28a(+) derivative carrying ramB (rv0465c) gene; kanR	This work 	
pUT18C	Bacterial two-hybrid vector encoding the T18 subunit of the cyaA catalytic domain from Bordetella pertussis; ampR	[34]	
pUT18CprpRmt	pUT18C derivative encoding a T18-PrpRMt fusion protein; used directly in bacterial two-hybrid assays; ampR	This work 	
pKT25	Bacterial two-hybrid vector encoding the T25 subunit of the cyaA catalytic domain from B. pertussis; kanR	[34]	
pKT25prpRmt	pKT25 derivative encoding a T25-PrpRMt fusion protein; used directly in bacterial two-hybrid assays; kanR	This work	
p2NIL	Recombination vector used in targeted gene replacement; kanR	[31]	
pGOAL17	Gene replacement vector containing a PacI cassette encoding lacZ and sacB screening genes; ampR 	[31]	
p2NILÄprpRmt_1	p2NIL derivative carrying a 5' prpRmt upstream region (1,604 bp) and the first 198 bp of the prpRmt gene; kanR	This work	
p2NILÄprpRmt_1+2	p2NILÄprpRmt_1 derivative carrying the 3' part of the prpRmt gene (212 bp) followed by 1457 bp of the prpRmt downstream region; kanR	This work	
p2NILÄprpRmt_OK	p2NILÄprpRmt_1+2 derivative containing a PacI screening cassette from pGOAL17; final construct used to unmarked deletion of prpRmt gene on M. tuberculosis chromosome; kanR	This work	
pMV306	Mycobacterial integrating vector; kanR	Med-Immune Inc.	
pMV306prpRmt	pMV306 derivative carrying a 5' prpRmt upstream region (300 bp) and the prpRmt gene; kanR	This work	
