# Supplementary material for: A Novel Role of the PrpR as a Transcription Factor Involved in the Regulation of Methylcitrate Pathway in Mycobacterium tuberculosis
Source: PLoS One. 2012 Aug 16;7(8):e43651. doi: 10.1371/journal.pone.0043651 (PMC3420887; doi:10.1371/journal.pone.0043651)
Supplement: Table S5 — Oligonucleotides (primers) used in DNA cloning and qPCR. (DOC) [file pone.0043651.s011.doc]

Table S5. Oligonucletides (primers) used in DNA cloning and qPCR

| **Oligonucleotide** | **Sequence (5`-3`) and features** | **Application** |
| --- | --- | --- |
| Rv1129_Fw | GGTCTAGAGGGATCCATGACGCGGAGTAATGTCTTAC (*Xba*I, *Bam*HI) | Amplification of  *M. tuberculosis prpR* (*rv1129c*) gene |
| Rv1129_Rv | GGGGTACC CTCGAGTCAAACCGATTGCTCGGTCGA (*Kpn*I, *Xho*I) |
| RamB_Fw | CGGGTACCGGGATCCGTGTCCAAGACCTACGTCGGCT (*Kpn*I, *Bam*HI) | Amplification of  *M. tuberculosis* *ramB* (*rv0465c*) gene |
| RamB_Rv | CGGAATTC AAGCTTTCATAGTTGTTTCACCAGATACGGG (*Eco*RI, *Hin*dIII) |
| **Targeted replacement of *M. tuberculosis prpR* gene** | | |
| 1Fw_delta1129 | GGAGTACTCGGTCATCGAAAGATTCCTGGTG (*Sca*I) | Unmarked *prpR* deletion on *M. tuberculosis* chromosome |
| 2Rv_delta1129 | GGAAGCTTTTAGGTAAGCAGAAGCAGCACGGG (*Hin*dIII) |
| 3Fw_delta1129 | GGAAGCTTGCTGCGACCTCGCGCACGCC (*Hin*dIII) |
| 4Rv_delta1129 | GGTTAATTAACTACGGGTCGCCTTCGTCGTC (*Pac*I) |
| 1129komp_Fw | GGAAGCTTGGTGGCGGGCCACGGTGAC (*Hin*dIII) | Complementation of *prpR* on *M. tuberculosis* Δ*prpR* chromosome |
| 1129komp_Rv | GGATCGATCACCACCGAGATGCCG (*Cla*I) |
| **qPCR analysis of *M. tuberculosis* genes expression levels** | | |
| RT_1129_Fw | GAGGGATACCGTTCATCTTCGT | *prpR* (*rv1129c*) transcription |
| RT_1129_Rv | GCGGACTGTCGCTTTGAGA |
| RT_MtprpD_Fw | TGAAGTGATCGTGGACGAACTG | *prpD* (*rv1130*) transcription |
| RT_MtprpD_Rv | CTGTTCAACGGGTTCCACTACA |
| RT_MtprpC_Fw | ATTCGTTGACCTACCGGGGATA | *prpC* (*rv1131*) transcription |
| RT_MtprpC_Rv | CAGCATCGAGCGGTCCAC |
| RT_Mticl_Fw | CAGCACATCCGCACTTTGAC | *icl1* (*rv0467*) transcription |
| RT_Mticl_Rv | ATCACCACCGTGGGAACATC |
| RT_MtramB_Fw | CCTGCCCGCTGTGGAA | *ramB* (*rv0465c*) transcription |
| RT_MtramB_Rv | GGCGATTTGCACCAAGATCT |
| RT_MtkstR_Fw | CCAGCGGCTGAACTTTATGG | *kstR* (*rv3574*) transcription |
| RT_MtkstR_Rv | TGTCATGGCCTCGGTGAGTA |
| RT_MtsigA_Fw | GGTGATTTCGTCTGGGATGAA | *sigA* (*rv2703*) transcription |
| RT_MtsigA_Rv | GCTACCTTGCCGATCTGTTTG |
| **Amplification of DNA fragments for EMSA and SPR** | | |
| p1129_Fw | GACGTCAACCGGATCGGCAGC | *prpDR* (*rv1130-1129c*) promoter region |
| p1129_Rv | GGCACCGGAAAACGTCCTCGA |
| biot-p1129_Rv | biot-GGCACCGGAAAACGTCCTCGA  (5’ biotin labelled) |
| picl_Fw | GGGGGTTTACCTGCGGATTTGTCG | *ic1l* (*rv0467*) promoter region |
| picl_Rv | GGTGCTCCGCGCTCTTCGGGGT |
| pkstR_Fw | GGTGCTAACGATCAACCGTCAAGTC | *kstR* (*rv3574*) promoter region |
| pkstR_Rv | GGGTGCCTCCGATCCGAGCTCG |
| biot-pkstR_Rv | biot-GGGTGCCTCCGATCCGAGCTCG (5’ biotin labeled) |
| pramB_Fw | GGCGGTCGAACACGTCGGGGTG | *ramB* (*rv0465c*) promoter region |
| pramB_Rv | GGGCAGTTGGCGGACCCGCGA |
| biot-pramB_Rv | biot-GGGCAGTTGGCGGACCCGCGA  (5’ biotin labeled) |
| pmtrA_Fw | CTTGCGGTCTCTGCCGAGCTC | *mtrA* (*rv3246c*) promoter region (negative control in EMSA) |
| pmtrA_Rv | TGGTGTCCATGGTGTCACCACA |
| padpA_EmFw | TTCCGCGCACAGTTCCGCTTC | *S. coelicolor* *adpA* (*SCO2792*) promoter region (negative control in SPR) |
| biot-padpA_EmRv | biot-ACGTTTCCCCTCGGTCCTGCA  (5’ biotin labeled) |
| **Analysis of *prpRmt* transcription start site** | | |
| p1129map_Fw1 | CGCTACCTCCGGGTCGACGT | PCR-based mapping of *prpRmt* transcription start site on cDNA template |
| p1129map_Fw2 | CGTCGGCGCTGCGCCACGC |
| p1129map_Fw3 | GACCCTGAAAAAGCGTACTTTTGTG |
| p1129map_Fw3 Up1 | CCGGACCGCATGCATCAACAT |
| p1129map_Fw3 Up2 | AATCCGCACCATACGCAGCAC |
| p1129map_Fw3 Up3 | AGGACACCAACAAAAGACCCTG |
| p1129map_Fw3 Down1 | GTCCTGATCCGGCACATTTTGC |
| p1129map_Fw2 Up1 | GTGCACGGGAAATCGTCGGCG |
| p1129map_Fw2 Fw3Up1 | CGCCACGCCCGGACCGCAT |
| p1129map_Rv | CTCGAATACGTCCTAGCCACCG |
